# Association Between Opioid-Related Mortality and History of Surgical Procedure: A Population-Based Case-Control Study

**DOI:** 10.1097/AS9.0000000000000412

**Published:** 2024-04-05

**Authors:** Mhd Wasem Alsabbagh, Michael A. Beazely, Leona Spasik

**Affiliations:** * From the School of Pharmacy, Faculty of Science, University of Waterloo, Waterloo, ON, Canada.

**Keywords:** analgesics, mortality, operative procedure, opioids, opioid addiction, surgery, surgical procedures

## Abstract

**Objective:**

This study examined whether there is an association between opioid-related mortality and surgical procedures.

**Methods:**

A case-control study design using deceased controls compared individuals with and without opioid death and their exposure to common surgeries in the preceding 4 years. This population-based study used linked death and hospitalization databases in Canada (excluding Quebec) from January 01, 2008 to December 31, 2017. Cases of opioid death were identified and matched to 5 controls who died of other causes by age (±4 years), sex, province of death, and date of death (±1 year). Patients with HIV infection and alcohol-related deaths were excluded from the control group. Logistic regression was used to determine if there was an association between having surgery and death from an opioid-related cause by estimating the crude and adjusted odds ratios (ORs) with the corresponding 95% confidence interval (CI). Covariates included sociodemographic characteristics, comorbidities, and the number of days of hospitalization in the previous 4 years.

**Results:**

We identified 11,865 cases and matched them with 59,345 controls. About 11.2% of cases and 12.5% of controls had surgery in the 4 years before their death, corresponding to a crude OR of 0.89 (95% CI: 0.83–0.94). After adjustment, opioid mortality was associated with surgical procedure with OR of 1.26 (95% CI: 1.17–1.36).

**Conclusions:**

After adjusting for comorbidities, patients with opioid mortality were more likely to undergo surgical intervention within 4 years before their death. Clinicians should enhance screening for opioid use and risk factors when considering postoperative opioid prescribing.

## Abstract 2

Mini abstract:

Is there an association between opioid-related mortality and having a surgical procedure within 4 years before the date of death? This is a case-control study using linked death and hospitalization databases in Canada (excluding Quebec) from January 01, 2008 to December 31, 2017. Cases of opioid death were identified and matched to 5 controls who died of other causes. After adjusting for covariates, we found a positive association between a history of a surgical procedure in the 4 years before death and opioid-related mortality (odds ratio = 1.26, 95% confidence interval: 1.17–1.36). Screening for opioid use and risk factors when considering postoperative opioid prescribing is warranted.

## INTRODUCTION

Opioid-related mortality has risen in Canada and globally.^1,2^ In the second quarter of 2021 (between April and June), there were 1913 opioid toxicity deaths reported in Canada, representing an 80% increase from the same period in 2019.^3^ This was well-established even before the effects of the COVID pandemic in Canada,^4^ as shown by the increase in opioid-related mortality from 2000 (475 deaths) to 2017 (3290 deaths).^5^

Opioid diversion partly contributes to the opioid epidemic in Canada. In 2021, 7% of accidental opioid toxicity deaths in Canada were attributed to opioids of a pharmaceutical-only origin.^3^ This number has been declining in recent years as pharmaceutical-only origin represented 25% of accidental opioid toxicity deaths in 2018.^3^ While this decline is encouraging, it is important to consider that patients often transition to the use of illicit opioids after initial exposure to opioids through the health care system.^6^ A study showed that 37.8% of people who had an active opioid prescription at the time of opioid-related death were identified as having nonprescribed opioids in their system based on toxicology results.^7^ Additionally, 80% of people who went to the emergency department for opioid toxicity in 2016 had been prescribed an opioid in the preceding 3 years.^7^

Postsurgical pain was identified as the second most common indication for opioid initiation for pain management in Ontario (17.4% of patients), second only to dental pain (23.2% of patients).^8^ As such, it is important to consider how surgical interventions may be linked to the rising opioid epidemic in Canada as postoperative prescriptions may provide an initial gateway to opioid use.^9^ Despite the literature relating surgery to prolonged opioid use, the association between opioid-related mortality and surgery has not been thoroughly investigated. Some studies have examined postsurgical clinical consequences that may be related to prolonged opioid use, such as hospital readmission rates, emergency department visits, postoperative complications, all-cause mortality, and mean length of hospital stay.^10–16^ However, these clinical consequences were not directly correlated with opioid use. For example, Kurteva et al^11^ found that 45% of postoperative cancer patients had an emergency department visit or hospital readmission primarily due to surgical complications or cancer-related reasons, not opioid overdose. On the other hand, the duration of follow-up after surgery in previous studies, which ranged from 3 months to 2 years,^10,12,17–20^ may have limited the ability to show an association with opioid-related outcomes.

Determining whether there is an association between surgery and opioid death can help provide clear guidance to medical professionals regarding the postoperative prescribing and monitoring practices for patients with postoperative opioid use. This study aimed to examine the association between surgical procedures and opioid-related mortality in a Canadian population. We hypothesized that there would be significantly higher odds of surgical intervention among individuals who died from an opioid-related cause than among those who died of other causes.

## METHODS

### Study Design

A case-control design (dead controls) was used to compare individuals with opioid-related mortality (cases) to individuals who died due to any other reason (the controls). Deceased controls were used to provide comparable accuracy and data quality when comparing exposure between the case and control groups.^21^

### Data Sources

Data sources include the deidentified Canadian Institute of Health Information Discharge Abstract Database (DAD) from the fiscal year (FY) 2004/2005 to FY2017/2018 linked to the Canadian Vital Statistics Deaths Database from 2008 to 2017. The DAD contains data on inpatient hospital discharges and day surgery interventions from all Canadian acute care institutions (excluding Quebec), including transfers, sign-outs, and in-hospital deaths.^22^ Diagnoses in DAD were coded using the International Classification of Diseases, 9th (until FY2006/2007) and 10th editions (ICD-9 and ICD-10), while medical interventions during hospitalizations were coded via the Canadian Classification of Health Interventions.^22^ The Canadian Vital Statistics Deaths Database is an administrative database that captures all deaths in Canada, with demographic and medical attributes. The main and additional causes of death were coded using ICD-10.^23^ Linkage was performed by Statistics Canada deterministically using the date of birth, postal code, sex, and health insurance number of patients and deceased within a highly secure linkage environment.

#### Cases and Controls

Cases included individuals who died of an opioid cause, while controls were individuals who died of any other reason in Canada between January 01, 2008 and December 31, 2017. Individuals who died in Quebec or did not have a recorded province of death were excluded. Controls were excluded if they died of HIV- or alcohol-related death; 2 disorders that may be associated with higher rates of opioid use than the general population, which could confound the outcome.^24^ A sensitivity analysis was conducted to determine whether including controls with HIV- or alcohol-related deaths would change the results.

### Measurements

#### Opioid Mortality

Opioid-related mortality was identified using a modified version of an algorithm developed by the Centers for Disease Control and Prevention plus heroin (Supplemental Appendix 1, see http://links.lww.com/AOSO/A314).^5,24^ The algorithm defines opioid-related mortality as death directly related to opioid poisoning, with at least one opioid contributing to multiple causes of death.^5,24^ The algorithm has a positive predictive value of 90% and a sensitivity of 75%.^5,24^

#### Surgical Procedure

The exposure of interest was having surgery in the linked DAD within 4 years before death. We examined a 4-year surgical history to have a long follow-up period to show any association with mortality. Surgeries included the most common surgeries in Canada, as reported by Canadian Institute of Health Information, encompassing close to 90% of all surgeries that are performed in Canada: cataract and other lenses, colectomy, coronary artery angioplasty, coronary artery bypass graft, disc, eardrum and/or mastoid repair, fractures, hernia, hip replacement, hysterectomy, knee replacement, open control of bleeding, drainage, removal of device and inspection, abdominal cavity, pacemaker insertion, prostatectomy, removal of appendix, removal of gallbladder, repair of nasal cartilage and/or nose, repair of retina, sterilization, and tonsillectomy. These were identified using Canadian Classification of Health Interventions codes (Supplemental Appendix 2, see http://links.lww.com/AOSO/A314).

#### HIV/AIDs or Alcohol Use Disorders

The codes used to identify HIV/AIDS or alcohol use disorders as causes of death are available in Supplemental Appendix 3, see http://links.lww.com/AOSO/A314.^25^

#### Covariates

The covariates examined included sociodemographic characteristics (age, sex, marital status, and income quintile), comorbidities, and the number of days of hospitalization in the previous 4 years. Age categories included <40, 40–59, and 60 years of age and over. Marital status categories included married or unmarried (single, widowed, divorced, separated, and unknown).Income was defined as the deceased's neighborhood median household income quintile within the province at the forward sortation area (FSA) level. The FSA refers to the first 3 characters of the patient’s postal code, which describe the geographical area, classification of urban or rural, and a description of the area as a city, town, or other.^26^ Comorbidities were identified in the 4 years before the death date using hospitalizations and included alcohol-related diagnosis, chronic lung disease (asthma or chronic obstructive pulmonary disease), chronic liver disease, chronic kidney disease, fibromyalgia, heart disease, lower back pain, mental health, opioid poisoning/opioid use disorder, osteoarthritis, posttraumatic pain, and rheumatoid arthritis as identified using ICD-9 and ICD-10 codes in any diagnosis field (Supplemental Appendix 4, see http://links.lww.com/AOSO/A314).^27,28^ These factors may be related to increased opioid mortality.^27^ The number of days of hospitalizations in the previous 4 years was categorized to 0–46, 46–92, 93–138, and +139.

### Matching

Cases were matched to 5 controls if their age at death was within ±4 years, they were the same sex, died in the same province, and their date of death was within ±365 days of cases. These criteria were intended to reduce the differences in the number of surgical interventions between cases and controls.

### Data Analysis

Descriptive statistics were used to describe the differences in exposure to surgery and the covariates between cases and controls. χ^2^ and *t* tests were used to examine the statistical significance of differences. Then, a logistic regression was used to identify if there was an association between having surgery and death of an opioid-related cause by estimating the odds ratio (OR) with the corresponding 95% confidence interval (CI). We first fit a reduced model with exposure only (ie, crude OR estimation), then a full model with exposure and all covariates (ie, adjusted OR [aOR] estimation). SAS (version 9.4) was used for the data analysis. All predetermined covariates, selected based on prior studies,^27,28^ and clinical significance were entered into the model.^29^

We performed a subgroup analysis using 3 clinical and 2 socioeconomic status variables. First, we stratified the sample by having a hospitalization for opioid use disorder or acute poisoning, a hospitalization for alcohol-related reasons, or hospitalization for a mental health reason in the previous 4 years. On the other hand, we stratified patients by their marital status into single (including those who are divorced, separated, or with unknown status) and married. Additionally, we stratified the sample by the household income quintile for their residential area into the lowest quintile and the highest quintile.

For each stratifying variable, we estimated the crude and aORs for each of the 2 levels of this stratifying variable. In the adjusted model, we included all other covariates, except for the stratifying variable.

Data were accessed at the South-Western Ontario Research Data Centre and rounded to the nearest 5 (numbers less than 5 were masked) according to Statistics Canada disclosure control guidelines. Ethics approval was obtained from the University of Waterloo Research Ethics Board (REB no. 41558).

## RESULTS

We identified 11,865 cases of opioid-related mortality and matched them with 59,345 controls for a total sample of 71,210 individuals. The baseline characteristics are summarized in Tables 1 and 2.

**TABLE 1.** Baseline Characteristics of Categorical Variables

Characteristic	Case (n = 11,865)	%	Control (n = 59,345)	%	Total (N = 71,210)	*P*
Had surgical procedure in the previous 4 yr	1330	11.2	7400	12.5	8730	0.0002
Had alcohol-related diagnosis	545	4.6	3210	5.4	3750	0.0003
Had chronic kidney disease diagnosis	100	0.8	1620	2.7	1720	<0.0001
Had chronic lung disease diagnosis	540	4.6	2675	4.5	3220	0.8528
Had chronic liver disease diagnosis	315	2.7	5085	8.6	5400	<0.0001
Had fibromyalgia diagnosis	25	0.2	30	0.1	55	<0.0001
Had heart disease diagnosis	745	6.3	7035	11.9	7785	<0.0001
Had lower back pain diagnosis	625	5.3	2215	3.7	2840	<0.0001
Had mental health diagnosis	1710	14.4	6790	11.4	8500	<0.0001
Had hospitalization of opioid poisoning or opioid use disorder	1630	13.7	1260	2.1	2895	<0.0001
Had osteoarthritis diagnosis	215	1.8	465	0.8	680	<0.0001
Had rheumatoid arthritis diagnosis	30	0.3	140	0.2	170	0.8633
Total hospitalization days in previous 4 yr						<0.0001
0–46	10,950	92.3	49,075	82.7	60,025	
47–92	525	4.4	5855	9.9	6385	
93–138	180	1.5	2110	3.6	2295	
>139	205	1.7	2305	3.9	2515	
Marital status						<0.0001
Unmarried (single, widowed, divorced, separated, or unknown)	9795	82.6	37,870	63.8	47,665	
Married	2070	17.4	21,475	36.2	23,545	
Sex						1
Females	4145	34.9	20,720	34.9	24,865	
Males	7725	65.1	38,625	65.1	46,350	
Age						
<40 yr old	4140	34.9	20,220	34.1	24,365	<0.0001
40–59 yr old	6350	53.5	30,570	51.5	36,920	
60+	1380	11.6	8550	14.4	9930	
FSA income quintile:						<0.001
Missing	300	2.5	1010	1.7	1310	
Quintile 1	950	8	7140	12	8090	
Quintile 2	1505	12.7	8990	15.1	10,495	
Quintile 3	2475	20.9	12,900	21.7	15,380	
Quintile 4	3000	25.3	15,325	25.8	18,325	
Quintile 5	3635	30.6	13,980	23.6	17,615	
Province of death						1
Alberta	2000	16.9	9990	16.8	11,990	
British Columbia	2830	23.9	14,155	23.9	16,990	
Manitoba	455	3.8	2285	3.9	2740	
New Brunswick, Newfoundland, Prince Edward Island, Territories	165	1.4	825	1.4	990	
Nova Scotia	475	4	2360	4	2835	
Ontario	5540	46.7	27,695	46.7	33,230	
Saskatchewan	410	3.5	2035	3.4	2445	

**TABLE 2.** Baseline Characteristics of Continuous Variables

Characteristic	Mean	SD	Mean	SD	Mean	*P*
Age (yr)	44	13.8	45	14.5	45	<0.01
No. hospitalization days in previous 4 yr	13	46.5	78	11,982	67	0.1
No. hospitalizations in previous 4 yr	2	3.2	3	4.1	3	<0.01

### Baseline Characteristics

Almost two-thirds of the sample (65.1%) was male, and 46.7% of deaths happened in Ontario. The mean age of the patients was 44 years and that of the controls was 45 years. About 17.4% of cases identified as married compared with 36.2% of controls. Residing in lowest income areas was more common in cases, with 30.6% of cases in FSA quintile 5 (the lowest income quintile) versus 23.6% of controls. The mean number of hospitalizations in the previous 4 years was 2 and 3 for cases and controls, respectively. The mean number of days spent hospitalized in the past 4 years was 13 and 78 days for cases and controls, respectively.

A total of 1330 cases (11.2%) and 7400 controls (12.5%) underwent at least one surgical procedure in the 4 years preceding their death. There were more patients who had lower back pain (5.3% of cases vs 3.7% of controls), osteoarthritis (1.8% of cases vs 0.8% of controls), chronic lung disease (4.6% of cases vs 4.5% of controls), fibromyalgia (0.2% of cases vs 0.1% of controls), rheumatoid arthritis (0.3% of cases vs 0.2% of controls), and mental health diagnoses (14.4% of cases vs 11.4% of controls). Additionally, cases had a higher hospitalization for opioid poisoning or opioid use disorder (13.7% of cases vs 2.1 % of controls).

Controls had higher alcohol-related diagnosis hospitalizations (5.4% of controls vs 4.6% of cases), chronic kidney disease (2.7% of controls vs 0.8% of cases), chronic liver disease (8.6% of controls vs 2.7% of cases), and heart disease (11.9% of controls vs 6.3% of cases).

### Logistic Regression

When fitting a crude logistic regression, the odds of having surgery in the 4 years before death among cases was significantly lower with a crude OR of 0.89 (95% CI: 0.8–0.94). After adjusting for covariates, the odds of having surgery was higher with an aOR of 1.26 (95% CI: 1.17–1.36).

The results of covariate logistic regression analysis are summarized in Table 3. Covariate analysis revealed that cases had a higher odds of hospitalizations in the 4 years preceding death with opioid poisoning or opioid use disorder (OR = 10.79, 95% CI: 9.85–11.83), fibromyalgia diagnosis (OR = 2.59, 95% CI: 1.38–4.89), osteoarthritis (OR = 2.56, 95% CI: 2.12–3.09), alcohol-related diagnosis (OR = 1.16, 95% CI: 1.03–1.30), chronic lung disease diagnosis (OR = 1.13, 95% CI: 1.01–1.26), lower back pain diagnosis (OR = 1.50, 95% CI: 1.35–1.66), and mental health diagnosis (OR = 1.30, 95% CI: 1.21–1.39). On the other hand, cases had significantly lower odds of having a chronic kidney disease diagnosis (OR = 0.49, 95% CI: 0.39–0.61), chronic liver disease diagnosis (OR = 0.31, 95% CI: 0.28–0.36), and heart disease diagnosis (OR = 0.47, 95% CI: 0.43–0.52).

**TABLE 3.** Logistic Regression Analysis

Characteristic	Odds Ratio (95% CI)
Surgical procedure (crude)	0.89 (0.83–0.94)
Surgical procedure (adjusted)	1.26 (1.17–1.36)
Alcohol-related diagnosis hospitalization (yes vs no)	1.16 (1.03–1.30)
Chronic kidney disease diagnosis hospitalization (yes vs no)	0.49 (0.39–0.61)
Chronic lung disease diagnosis hospitalization (yes vs no)	1.13 (1.01–1.26)
Chronic liver disease diagnosis hospitalization (yes vs no)	0.31 (0.28–0.36)
Fibromyalgia diagnosis hospitalization (yes vs no)	2.59 (1.38–4.89)
Heart disease diagnosis hospitalization (yes vs no)	0.47 (0.43–0.52)
Lower back pain diagnosis hospitalization (yes vs no)	1.50 (1.35–1.66)
Mental health diagnosis hospitalization (yes vs no)	1.30 (1.21–1.39)
Hospitalization for opioid poisoning or opioid use disorder (yes vs no)	10.79 (9.85–11.83)
Osteoarthritis hospitalization (yes vs no)	2.56 (2.12–3.09)
Rheumatoid arthritis diagnosis hospitalization (yes vs no)	1.24 (0.78–1.95)
Had <7 hospitalizations in last 4 yr (vs 0)	0.41 (0.38–0.45)
Had <10 hospitalizations in last 4 yr (vs 0)	0.29 (0.26–0.33)
Had <244 hospitalizations in last 4 yr (vs 0)	0.27 (0.23–0.31)
Unmarried (single, widowed, divorced, separated, or unknown marital status) vs married	2.39 (2.26–2.52)
Males vs females	0.92 (0.88–0.97)
FSA quintile (2 vs 1)	1.19 (1.09–1.30)
FSA quintile (3 vs 1)	1.30 (1.20–1.41)
FSA quintile (4 vs 1)	1.30 (1.20–1.4)
FSA quintile (5 vs 1)	1.59 (1.47–1.73)
Age group (60+ vs <40)	1.15 (1.07–1.24)
Age group (40–59 vs <40)	1.32 (1.26–1.39)
Alberta vs Saskatchewan	0.96 (0.84–1.09)
British Columbia vs Saskatchewan	0.97 (0.85–1.09)
Manitoba vs Saskatchewan	0.98 (0.84–1.15)
New Brunswick, Newfoundland, Prince Edward Island, Territories vs Saskatchewan	1.06 (0.86–1.31)
Nova Scotia vs Saskatchewan	1.05 (0.90–1.23)
Ontario vs Saskatchewan	1.01 (0.89–1.13)

### Sensitivity Analysis

There was a slight reduction in the aOR of having a surgical procedure when including HIV- and alcohol-related deaths in the control pool (OR = 1.19, 95% CI: 1.10–1.28). Additionally, the OR of the covariates of hospitalizations with alcohol-related diagnosis and chronic lung disease became insignificant, while hospitalizations with fibromyalgia diagnosis, mental health diagnosis, opioid poisoning, or opioid use disorder remained significant, albeit with lower ORs. All the other results remained relatively similar between the main and sensitivity analyses. Table 4 presents the results of the sensitivity analysis.

**TABLE 4.** Sensitivity Analysis (Including HIV/Alcohol Deaths in Controls Pool)

Characteristic	Odds Ratio (95% CI)
Surgical procedure (adjusted)	1.19 (1.10–1.28)
Alcohol-related diagnosis	1.07 (0.95–1.20)
Alcohol-related diagnosis hospitalization (yes vs no)	0.54 (0.43–0.68)
Chronic kidney disease diagnosis hospitalization (yes vs no)	1.11 (0.99–1.24)
Chronic lung disease diagnosis hospitalization (yes vs no)	0.31 (0.27–0.36)
Chronic liver disease diagnosis hospitalization (yes vs no)	2.09 (1.17–3.74)
Fibromyalgia diagnosis hospitalization (yes vs no)	0.48 (0.44–0.52)
Heart disease diagnosis hospitalization (yes vs no)	1.53 (1.38–1.71)
Lower back pain diagnosis hospitalization (yes vs no)	1.31 (1.23–1.41)
Mental health diagnosis hospitalization (yes vs no)	10.37 (9.48–11.34)
Hospitalization for opioid poisoning or opioid use disorder (yes vs no)	2.44 (2.03–2.93)
Osteoarthritis hospitalization (yes vs no)	1.21 (0.78–1.90)
Unmarried (single, widowed, divorced, separated, or unknown marital status) vs married	2.35 (2.22–2.48)
Had <7 hospitalizations in last 4 yr	0.41 (0.38–0.45)
Had <10 hospitalizations in last 4 yr	0.28 (0.25–0.32)
Had <244 hospitalizations in last 4 yr	0.28 (0.24–0.32)
Males vs females	0.92 (0.88–0.96)
FSA quintile (2 vs 1)	1.22 (1.11–1.33)
FSA quintile (3 vs 1)	1.31 (1.20–1.42)
FSA quintile (4 vs 1)	1.31 (1.21–1.43)
FSA quintile (5 vs 1)	1.57 (1.45–1.71)
Age group (60+ vs <40)	1.16 (1.08–1.25)
Age group (40–59 vs <40)	1.33 (1.27–1.40)
Alberta vs Saskatchewan	0.96 (0.85–1.09)
British Columbia vs Saskatchewan	0.97 (0.86–1.10)
Manitoba vs Saskatchewan	1.00 (0.86–1.17)
New Brunswick, Newfoundland, Prince Edward Island, Territories vs Saskatchewan	1.12 (0.91–1.38)
Nova Scotia vs Saskatchewan	1.04 (0.89–1.21)
Ontario vs Saskatchewan	1.02 (0.90–1.14)

### Subgroup Analysis

When we performed the subgroup analysis (Supplemental Appendix 5, see http://links.lww.com/AOSO/A314), we found that the overall estimation was consistent among deceased individuals who had a previous hospitalization for an opioid-related diagnosis, an alcohol-related diagnosis, or a mental health diagnosis. However, for deceased individuals who did not have a mental health-related hospitalization in the previous 4 years, the association between dying of an opioid overdose and having surgery in the previous 4 years was even more pronounced. On the socioeconomic status side, being single or being from the lowest socioeconomic status made the association more consistent with the overall point estimates.

## DISCUSSION

This population-based case-control study used linked hospitalization and death databases between 2004 and 2017 and aimed to examine whether there is a higher exposure to common surgical procedures in the Canadian population (excluding Quebec) among individuals who died of an opioid-related cause in comparison to individuals who died from other causes. Exposure to surgery was lower among cases in the crude analysis with OR of 0.89 (95% CI: 0.83–0.94). However, after controlling for covariates of comorbidities, socioeconomic status, and demographics, individuals who died of an opioid-related cause had higher odds of having at least one of the most common surgeries in the 4 years before their death (aOR = 1.26, 95% CI: 1.17–1.36). Including HIV- or alcohol-related deaths in the control group yielded similar results, albeit a lower aOR of 1.19 (95% CI: 1.10–1.28). The results of this study showed that opioid-related mortality was associated with a surgical procedure in the previous 4 years.

Postsurgical pain is a common indication for opioid prescribing.^30^ However, postsurgical opioid prescriptions are not always provided in a manner that guarantees patients safety.^31^ Almost 1 in 4 patients were found to be prescribed a “high-risk” opioid – defined as coprescribing of benzodiazepines, more than one prescriber, high opioid daily doses, or long-acting opioids.^32^ New persistent use of opioids after surgery was found to be associated with higher mortality and worse health outcomes.^20^ Available research from Canada on this topic has examined how postoperative opioid prescribing impacts continued opioid use after specific types of surgery.^10–12,18,19,33^ For example, Welk et al^10^ found that there were 43% higher odds of new, persistent opioid use after minor urologic surgery in opioid-naive men who had postoperative exposure to opioids than those who did not. The secondary outcome demonstrated an increased likelihood of emergency room visits or hospital admissions for opioid overdose in men who filled an opioid prescription 5 days after their procedure.^10^ Literature also suggests that patients who undergo certain surgeries, and are preexisting opioid users, have an increased risk of prolonged opioid use.^34–37^ Hinther et al^34^ examined patients who underwent primary head and neck surgical resection with free flap reconstruction and found that preoperative opioid use was significantly correlated to prolonged postoperative opioid use, with a higher prevalence rate than the opioid-naive group.

The findings of the crude analysis can be explained by several factors. First, the mean age was 44 years for cases and 45 years for the controls due to matching. In Canada, the life expectancy at birth between 2007 and 2009 was 81.1 years, indicating that individuals in our study died at a much younger age than the average Canadian.^28^ The controls may have more comorbidities than the average Canadian as well; for example, 8.3% of Canadians are estimated to live with heart disease compared with 11.9% of controls in our cohort, despite the age difference.^38^ Similarly, more controls had chronic kidney disease and chronic liver disease than cases. Controls spent 78 days hospitalized in the 4 years before their death, while cases spent only 13 days hospitalized. Since surgery is often offered to patients with advanced disease states, the poorer health status of individuals with nonopioid mortality may explain the increased likelihood of having surgery in the controls before adjustment. Nevertheless, after adjusting for comorbidities and other characteristics, there were significantly increased odds of exposure to surgery among individuals who died of opioid-related mortality compared with those who died of other causes. Our study adds to previous literature that has established a relationship between specific types of surgery and prolonged opioid use afterward.^10,34^ Previous research either failed to find an association between exposure to opioids after surgery and opioid-related mortality,^11,13,14^ or found a positive association between high opioid use and mortality.^20^ We found that exposure to surgery alone is associated with opioid-related mortality. This is because surgery may be a surrogate for opioid prescription, which translates to an increased risk of opioid-related mortality, as patients are often exposed to opioids through the healthcare system before transitioning to illicit opioids.

Understanding that exposure to surgery increases in patients with opioid mortality is a critical finding for surgical practice. Clinicians screening for opioid use risk factors before surgical interventions or postoperative opioid prescribing is recommended.^39^ As such, our results illustrate the importance and need of implementing multidisciplinary stewardship interventions to promote appropriate opioid prescribing after surgery.^40^ Furthermore, our results may encourage clinicians to offer patients at high risk of opioid misuse with alternative pain therapies postsurgery. However, it is important to consider that inappropriate pain management can be counterproductive to surgical rehabilitation and lead to complications such as chronic pain.^41^ This emphasizes the importance of diligent screening for opioid abuse potential and careful consideration of the benefits and risks of opioids in postsurgical pain management.^42^

### Strengths and Limitations

We have studied the association between opioid-related mortality and common surgeries (almost 90% of all surgeries) among all Canadians outside of Quebec over 10 years. However, this study has some limitations. First, the algorithm used to identify opioid mortality has a sensitivity of 75%, allowing some patients who had opioid-related mortality to be classified into the control group, which could drive results toward the null hypothesis.^5,24^ Additionally, the data sources may lack comprehensiveness due to nonresponses by some institutions and nonmandated submission of day surgery data to the DAD in Prince Edward Island, Nova Scotia, Ontario, and Alberta.^43^ However, this difference is not expected to be different between cases and controls. The data used for controlling comorbidities were recorded at acute care institutions, meaning that medical conditions may have been missed in the adjusted analysis if the patient did not present to the hospital. Finally, we were unable to assess opioid use after surgery. Therefore, it is difficult to determine whether surgery is the true cause of opioid addiction and overdose. We assessed only the most common surgeries performed, although certain surgeries may be associated with higher levels of pain and opioid use. This information would be helpful in providing a more accurate assessment of opioid abuse risk for specific types of surgeries.

## CONCLUSIONS

This study addresses gaps in the literature on the relationship between opioid-related mortality and exposure to surgery, which is a risk factor for prolonged opioid use. The results of this study demonstrated that patients with opioid mortality had higher odds of being exposed to surgery when accounting for comorbidities, demographics, and income. Future research should focus on practices after specific types of surgeries and their correlation with opioid mortality to better categorize this risk factor.

## ACKNOWLEDGMENTS

M.W.A. and M.B. conceived the study, and all authors contributed to the study design and methodology. M.W.A. and L.P. conducted all the analyses and wrote the first draft of the manuscript. All the authors contributed to and approved the final version of the manuscript.

The analysis presented in this article was conducted at the South-Western Ontario RDC (SWO-RDC), which is part of the Canadian Research Data Centre Network (CRDCN). The services and activities provided by the SWO-RDC are made possible by the financial or in-kind support of the SSHRC, the CIHR, the CFI, Statistics Canada, and University of Waterloo. The views expressed in this study do not necessarily represent the CRDCN’s or those of its partners.

Data were obtained through secured access to the South-Western Ontario Data Research Center at the University of Waterloo. No data sharing with the journal was offered.

## Supplementary Material


