# Supplementary material for: Association Between Opioid-Related Mortality and History of Surgical Procedure: A Population-Based Case-Control Study
Source: Ann Surg Open. 2024 Apr 5;5(2):e412. doi: 10.1097/AS9.0000000000000412 (PMC11191927; doi:10.1097/AS9.0000000000000412)
Supplement: Supplementary file 1 [file as9-5-e412-s001.pdf]

Appendix 1: Identifying Opioid-Related Mortality for Selection of Cases[2]

Cases were selected if their death was directly caused by opioid poisoning, as represented by the codes in Column 1, or if they had at least one opioid contributing to multiple causes of death (Column 2).[2] The cause of death was coded using ICD-10 codes.

The Modified Centers for Disease Control plus heroin algorithm.

**Direct Drug Poisoning Codes (Column 1)**	**Opioid Contributor Codes (Column 2)**
X40 – X49 (unintentional)	T40.1 (heroin)
X60 – X69 (intentional)	T40.2 (other opioids)
X85 – X90 (homicide poisoning)	T40.3 (methadone)
Y10 – Y19 (undetermined intent)	T40.4 (other synthetic narcotics)
	T40.6 (other and unspecified narcotics)

Appendix 1: Identification of Exposure to Surgical Procedure

Classification of Health Interventions (CCI) codes were used to identify patients who underwent common surgical procedures 4 years before their death.

Surgical intervention	CCI Code (version 2018)	Description
Caesarean section delivery	5MD60AA	Cesarean section, without instrumentation lower segment transverse incision
Caesarean section delivery	5MD60CB	Cesarean section, with use of both vacuum and forceps cesarean hysterectomy
Caesarean section delivery	5MD60CC	Cesarean section, with use of both vacuum and forceps classical section [vertical incision in upper segment]
Caesarean section delivery	5MD60CD	Cesarean section, with use of both vacuum and forceps extraperitoneal section
Caesarean section delivery	5MD60CE	Cesarean section, with use of both vacuum and forceps inverted T incision
Caesarean section delivery	5MD60CF	Cesarean section, with use of both vacuum and forceps low segment transverse incision
Caesarean section delivery	5MD60CG	Cesarean section, with use of both vacuum and forceps other type of cesarean section NEC
Caesarean section delivery	5MD60JW	Cesarean section, with use of forceps lower segment transverse incision
Caesarean section delivery	5MD60JX	Cesarean section, with use of vacuum lower segment transverse incision
Caesarean section delivery	5MD60JY	Cesarean section, without instrumentation classical section [vertical incision in upper segment]
Caesarean section delivery	5MD60JZ	Cesarean section, with use of forceps classical section [vertical incision in upper segment]
Caesarean section delivery	5MD60KA	Cesarean section, with use of vacuum classical section [vertical incision in upper segment]
Caesarean section delivery	5MD60KB	Cesarean section, without instrumentation extraperitoneal section
Caesarean section delivery	5MD60KC	Cesarean section, with use of forceps extraperitoneal section
Caesarean section delivery	5MD60KD	Cesarean section, with use of vacuum extraperitoneal section
Caesarean section delivery	5MD60KE	Cesarean hysterectomy without instrumentation
Caesarean section delivery	5MD60KF	Cesarean laparotomy (for abdominal pregnancy) without instrumentation
Caesarean section delivery	5MD60KG	Cesarean section, without instrumentation inverted T incision
Caesarean section delivery	5MD60KT	Cesarean section, without instrumentation other type of Cesarean section NEC
Caesarean section delivery	5MD60RA	Cesarean section, with use of forceps inverted 'T' incision
Caesarean section delivery	5MD60RB	Cesarean section, with use of vacuum inverted T incision
Caesarean section delivery	5MD60RC	Cesarean hysterectomy with use of forceps

Caesarean section delivery	5MD60RD	Cesarean hysterectomy with use of vacuum
Caesarean section delivery	5MD60RE	Cesarean laparotomy (for abdominal pregnancy) with use of forceps
Caesarean section delivery	5MD60RF	Cesarean laparotomy (for abdominal pregnancy) with use of vacuum
Caesarean section delivery	5MD60RG	Cesarean section, with use of forceps other type of Cesarean section NEC
Caesarean section delivery	5MD60RH	Cesarean section, with use of vacuum other type of Cesarean section NEC
Cataract and other lens surgery	1CL53LALM	Implantation of internal device, lens of folded posterior chamber lens prosthesis using open approach
Cataract and other lens surgery	1CL53LALN	Implantation of internal device, lens of rigid posterior chamber lens prosthesis using open approach
Cataract and other lens surgery	1CL53LALO	Implantation of internal device, lens of intraocular telescope using open approach
Cataract and other lens surgery	1CL53LALP	Implantation of internal device, lens of anterior chamber lens prosthesis using open approach
Cataract and other lens surgery	1CL53LALR	Implantation of internal device, lens of intraocular lens prosthesis [iris, sulcus fixated] using open approach
Cataract and other lens surgery	1CL59LAAG	Destruction, lens using open approach and laser
Cataract and other lens surgery	1CL59WK	Destruction, lens using incisional technique
Cataract and other lens surgery	1CL89NP	Excision total, lens extracapsular lens extraction NEC with no insertion of lens prosthesis
Cataract and other lens surgery	1CL89NPLM	Excision total, lens extracapsular lens extraction NEC with posterior chamber folded lens prosthesis inserted
Cataract and other lens surgery	1CL89NPLN	Excision total, lens extracapsular lens extraction NEC with posterior chamber rigid lens prosthesis inserted
Cataract and other lens surgery	1CL89NPLP	Excision total, lens extracapsular lens extraction NEC with anterior chamber lens prosthesis inserted
Cataract and other lens surgery	1CL89NPLR	Excision total, lens extracapsular lens extraction NEC with other lens prosthesis insertion (e.g. into ciliary sulcus or iris)
Cataract and other lens surgery	1CL89NV	Excision total, lens intracapsular lens extraction with no insertion of lens prosthesis
Cataract and other lens surgery	1CL89NVLP	Excision total, lens intracapsular lens extraction with anterior chamber lens prosthesis inserted
Cataract and other lens surgery	1CL89NVLR	Excision total, lens intracapsular lens extraction with other lens prosthesis insertion (e.g. into ciliary sulcus or iris)
Cataract and other lens surgery	1CL89VO	Excision total, lens phacoemulsification and Femtosecond laser with no insertion of lens prosthesis
Cataract and other lens surgery	1CL89VOLM	Excision total, lens phacoemulsification and Femtosecond laser with posterior chamber folded lens prosthesis inserted
Cataract and other lens surgery	1CL89VOLN	Excision total, lens phacoemulsification and Femtosecond laser with posterior chamber rigid lens prosthesis inserted
Cataract and other lens surgery	1CL89VOLO	Excision total, lens phacoemulsification and Femtosecond laser with intraocular telescope inserted
Cataract and other lens surgery	1CL89VOLP	Excision total, lens phacoemulsification and Femtosecond laser with anterior chamber lens prosthesis inserted
Cataract and other lens surgery	1CL89VOLR	Excision total, lens phacoemulsification and Femtosecond laser with other lens prosthesis insertion (e.g. into ciliary sulcus or iris)

Cataract and other lens surgery	1CL89VR	Excision total, lens phacoemulsification with no insertion of lens prosthesis
Cataract and other lens surgery	1CL89VRLM	Excision total, lens phacoemulsification with posterior chamber folded lens prosthesis inserted
Cataract and other lens surgery	1CL89VRLN	Excision total, lens phacoemulsification with posterior chamber rigid lens prosthesis inserted
Cataract and other lens surgery	1CL89VRLO	Excision total, lens phacoemulsification with intraocular telescope inserted
Cataract and other lens surgery	1CL89VRLP	Excision total, lens phacoemulsification with anterior chamber lens prosthesis inserted
Cataract and other lens surgery	1CL89VRLR	Excision total, lens phacoemulsification with other lens prosthesis insertion (e.g. into ciliary sulcus or iris)
Colectomy	1NM87DE	Excision partial, large intestine endoscopic [laparoscopic, laparoscopic-assisted, hand-assisted] approach colorectal anastomosis technique
Colectomy	1NM87DF	Excision partial, large intestine endoscopic [laparoscopic, laparoscopic-assisted, hand-assisted] approach colocolostomy anastomosis technique
Colectomy	1NM87DN	Excision partial, large intestine endoscopic [laparoscopic, laparoscopic-assisted, hand-assisted] approach enterocolostomy anastomosis technique
Colectomy	1NM87DX	Excision partial, large intestine endoscopic [laparoscopic, laparoscopic-assisted, hand-assisted] approach stoma formation and distal closure
Colectomy	1NM87DY	Excision partial, large intestine endoscopic [laparoscopic, laparoscopic-assisted, hand-assisted] approach stoma formation with creation of mucous fistula
Colectomy	1NM87GB	Excision partial, large intestine endoscopic [laparoscopic, laparoscopic-assisted, hand-assisted] approach special excisional (segmental) technique without anastomosis
Colectomy	1NM87RD	Excision partial, large intestine open approach colorectal anastomosis technique
Colectomy	1NM87RE	Excision partial, large intestine open approach enterocolostomy anastomosis technique
Colectomy	1NM87RN	Excision partial, large intestine open approach colocolostomy anastomosis technique
Colectomy	1NM87TF	Excision partial, large intestine open approach stoma formation and distal closure
Colectomy	1NM87TG	Excision partial, large intestine open approach stoma formation with creation of mucous fistula
Colectomy	1NM87WJ	Excision partial, large intestine open approach special excisional (segmental) technique without anastomosis
Colectomy	1NM89DF	Excision total, large intestine endoscopic [laparoscopic, laparoscopic-assisted, hand-assisted] approach ileorectal [endorectal, ileoproctostomy] anastomosis technique
Colectomy	1NM89DX	Excision total, large intestine endoscopic [laparoscopic, laparoscopic-assisted, hand-assisted] approach stoma formation with distal closure
Colectomy	1NM89GB	Excision total, large intestine endoscopic [laparoscopic, laparoscopic-assisted, hand-assisted] approach special excisional technique without anastomosis
Colectomy	1NM89RN	Excision total, large intestine open approach using ileorectal [endorectal, ileoproctostomy] anastomosis technique

Colectomy	1NM89TF	Excision total, large intestine open approach stoma formation with distal closure
Colectomy	1NM89WJ	Excision total, large intestine open approach special excisional technique without anastomosis
Colectomy	1NM91DE	Excision radical, large intestine endoscopic [laparoscopic, laparoscopic-assisted, hand-assisted] approach colorectal anastomosis technique
Colectomy	1NM91DF	Excision radical, large intestine endoscopic [laparoscopic, laparoscopic-assisted, hand-assisted] approach colocolostomy anastomosis technique
Colectomy	1NM91DN	Excision radical, large intestine endoscopic [laparoscopic, laparoscopic-assisted, hand-assisted] approach enterocolostomy anastomosis technique
Colectomy	1NM91DX	Excision radical, large intestine endoscopic [laparoscopic, laparoscopic-assisted, hand-assisted] approach stoma formation with distal closure
Colectomy	1NM91DY	Excision radical, large intestine endoscopic [laparoscopic, laparoscopic-assisted, hand-assisted] approach stoma formation with creation of mucous fistula
Colectomy	1NM91RD	Excision radical, large intestine open approach Colorectal anastomosis technique
Colectomy	1NM91RE	Excision radical, large intestine open approach Enterocolostomy anastomosis technique
Colectomy	1NM91RN	Excision radical, large intestine open approach Colocolostomy anastomosis technique
Colectomy	1NM91TF	Excision radical, large intestine open approach Stoma formation with distal closure
Colectomy	1NM91TG	Excision radical, large intestine open approach Stoma formation with creation of mucous fistula
Coronary artery angioplasty	1IJ50GQBD	Dilation, coronary arteries, without stent insertion percutaneous transluminal approach [e.g. with angioplasty alone] using balloon or cutting balloon dilator
Coronary artery angioplasty	1IJ50GQBF	Dilation, coronary arteries, without stent insertion percutaneous transluminal approach [e.g. with angioplasty alone] using laser (and balloon) dilator
Coronary artery angioplasty	1IJ50GQNR	Dilation, coronary arteries, with (endovascular) stent insertion percutaneous transluminal approach [e.g. with angioplasty alone] using (endovascular) stent only
Coronary artery angioplasty	1IJ50GQOA	Dilation, coronary arteries, with (endovascular) stent insertion percutaneous transluminal approach [e.g. with angioplasty alone] using balloon or cutting balloon dilator with (endovascular) stent
Coronary artery angioplasty	1IJ50GQOB	Dilation, coronary arteries, with (endovascular) stent insertion percutaneous transluminal approach [e.g. with angioplasty alone] using laser (and balloon) dilator with (endovascular) stent
Coronary artery angioplasty	1IJ50GQOD	Dilation, coronary arteries, without stent insertion percutaneous transluminal approach [e.g. with angioplasty alone] using ultrasound (and balloon) dilator
Coronary artery angioplasty	1IJ50GQOE	Dilation, coronary arteries, with (endovascular) stent insertion percutaneous transluminal approach [e.g. with angioplasty alone] using ultrasound (and balloon) dilator with (endovascular) stent
Coronary artery angioplasty	1IJ50GTBD	Dilation, coronary arteries, without stent insertion percutaneous transluminal approach with atherectomy

		[e.g. rotational, directional, extraction catheter, laser] using balloon or cutting balloon dilator
Coronary artery angioplasty	1IJ50GTBF	Dilation, coronary arteries, without stent insertion percutaneous transluminal approach with atherectomy [e.g. rotational, directional, extraction catheter, laser] using laser (and balloon) dilator
Coronary artery angioplasty	1IJ50GTOA	Dilation, coronary arteries, with (endovascular) stent insertion percutaneous transluminal approach with atherectomy [e.g. rotational, directional, extraction catheter, laser] using balloon or cutting balloon dilator with (endovascular) stent
Coronary artery angioplasty	1IJ50GTOB	Dilation, coronary arteries, with (endovascular) stent insertion percutaneous transluminal approach with atherectomy [e.g. rotational, directional, extraction catheter, laser] using laser (and balloon) dilator with (endovascular) stent
Coronary artery angioplasty	1IJ50GTOD	Dilation, coronary arteries, without stent insertion percutaneous transluminal approach with atherectomy [e.g. rotational, directional, extraction catheter, laser] using ultrasound (and balloon) dilator
Coronary artery angioplasty	1IJ50GTOE	Dilation, coronary arteries, with (endovascular) stent insertion percutaneous transluminal approach with atherectomy [e.g. rotational, directional, extraction catheter, laser] using ultrasound (and balloon) dilator with (endovascular) stent
Coronary artery angioplasty	1IJ50GUBD	Dilation, coronary arteries, without stent insertion percutaneous transluminal approach with thrombectomy using balloon or cutting balloon dilator
Coronary artery angioplasty	1IJ50GUBF	Dilation, coronary arteries, without stent insertion percutaneous transluminal approach with thrombectomy using laser (and balloon) dilator
Coronary artery angioplasty	1IJ50GUOA	Dilation, coronary arteries, with (endovascular) stent insertion percutaneous transluminal approach with thrombectomy using balloon or cutting balloon dilator with (endovascular) stent
Coronary artery angioplasty	1IJ50GUOB	Dilation, coronary arteries, with (endovascular) stent insertion percutaneous transluminal approach with thrombectomy using laser (and balloon) dilator with (endovascular) stent
Coronary artery angioplasty	1IJ50GUOD	Dilation, coronary arteries, without stent insertion percutaneous transluminal approach with thrombectomy using ultrasound (and balloon) dilator
Coronary artery angioplasty	1IJ50GUOE	Dilation, coronary arteries, with (endovascular) stent insertion percutaneous transluminal approach with thrombectomy using ultrasound (and balloon) dilator with (endovascular) stent
Coronary artery angioplasty	1IJ57GT	Extraction, coronary arteries using percutaneous transluminal approach for atherectomy
Coronary artery angioplasty	1IJ57GU	Extraction, coronary arteries using percutaneous transluminal approach for thrombectomy
Coronary artery bypass graft	1IJ76DAXXA	Bypass, coronary arteries endoscopic approach using autograft [e.g. saphenous]
Coronary artery bypass graft	1IJ76DAXXG	Bypass, coronary arteries endoscopic approach using pedicled flap [e.g. internal mammary, thoracic]

Coronary artery bypass graft	1IJ76DAXXN	Bypass, coronary arteries endoscopic approach using synthetic tissue (graft)
Coronary artery bypass graft	1IJ76DAXXQ	Bypass, coronary arteries endoscopic approach using combined sources of tissue (e.g. graft/pedicled flap)
Coronary artery bypass graft	1IJ76LAXXA	Bypass, coronary arteries open approach [sternotomy] using autograft [e.g. saphenous]
Coronary artery bypass graft	1IJ76LAXXG	Bypass, coronary arteries open approach [sternotomy] using pedicled flap [e.g. internal mammary, thoracic]
Coronary artery bypass graft	1IJ76LAXXN	Bypass, coronary arteries open approach [sternotomy] using synthetic tissue (graft)
Coronary artery bypass graft	1IJ76LAXXQ	Bypass, coronary arteries open approach [sternotomy] using combined sources of tissue (e.g. graft/pedicled flap)
Coronary artery bypass graft	1IJ76WKXXA	Bypass, coronary arteries minimal (beating heart keyhole) incisional technique [e.g. MIDCAB] using autograft [e.g. saphenous]
Coronary artery bypass graft	1IJ76WKXXG	Bypass, coronary arteries minimal (beating heart keyhole) incisional technique [e.g. MIDCAB] using pedicled flap [e.g. internal mammary, thoracic]
Coronary artery bypass graft	1IJ76WKXXN	Bypass, coronary arteries minimal (beating heart keyhole) incisional technique [e.g. MIDCAB] using synthetic tissue (graft)
Coronary artery bypass graft	1IJ76WKXXQ	Bypass, coronary arteries minimal (beating heart keyhole) incisional technique [e.g. MIDCAB] using combined sources of tissue [e.g. graft/pedicled flap]
Disc surgery	1SA75LLGXA	Fusion, atlas and axis anterior approach [anterolateral, retropharyngeal and transoral approaches] with autograft using device NEC
Disc surgery	1SA75LLGXK	Fusion, atlas and axis anterior approach [anterolateral, retropharyngeal and transoral approaches] with homograft [e.g. from bone bank] using device NEC
Disc surgery	1SA75LLGXN	Fusion, atlas and axis anterior approach [anterolateral, retropharyngeal and transoral approaches] with synthetic tissue [e.g. bone cement, paste, bioglass] using device NEC
Disc surgery	1SA75LLGXQ	Fusion, atlas and axis anterior approach [anterolateral, retropharyngeal and transoral approaches] with combined sources of tissue using device NEC
Disc surgery	1SA75LLKDA	Fusion, atlas and axis anterior approach [anterolateral, retropharyngeal and transoral approaches] with autograft using wire, staple, button, cabling, hook
Disc surgery	1SA75LLKDK	Fusion, atlas and axis anterior approach [anterolateral, retropharyngeal and transoral approaches] with homograft [e.g. from bone bank] using wire, staple, button, cabling, hook
Disc surgery	1SA75LLKDN	Fusion, atlas and axis anterior approach [anterolateral, retropharyngeal and transoral approaches] with synthetic tissue [e.g. bone cement, paste, bioglass] using wire, staple, button, cabling, hook
Disc surgery	1SA75LLKDQ	Fusion, atlas and axis anterior approach [anterolateral, retropharyngeal and transoral approaches] with combined sources of tissue using wire, staple, button, cabling, hook
Disc surgery	1SA75LLNWA	Fusion, atlas and axis anterior approach [anterolateral, retropharyngeal and transoral approaches] with autograft using screw, screw with plate, staple or pin [e.g. odontoid screw]

Disc surgery	1SA75LLNWK	Fusion, atlas and axis anterior approach [anterolateral, retropharyngeal and transoral approaches] with homograft [e.g. from bone bank] using screw, screw with plate, staple or pin [e.g. odontoid screw]
Disc surgery	1SA75LLNWN	Fusion, atlas and axis anterior approach [anterolateral, retropharyngeal and transoral approaches] with synthetic tissue [e.g. bone cement, paste, bioglass] using screw, screw with plate, staple or pin [e.g. odontoid screw]
Disc surgery	1SA75LLNWQ	Fusion, atlas and axis anterior approach [anterolateral, retropharyngeal and transoral approaches] with combined sources of tissue using screw, screw with plate, staple or pin [e.g. odontoid screw]
Disc surgery	1SA75LNGXA	Fusion, atlas and axis combined anterior and posterior approach with autograft using device NEC
Disc surgery	1SA75LNGXK	Fusion, atlas and axis combined anterior and posterior approach with homograft [e.g. from bone bank] using device NEC
Disc surgery	1SA75LNGXN	Fusion, atlas and axis combined anterior and posterior approach with synthetic tissue [e.g. bone cement, paste, bioglass] using device NEC
Disc surgery	1SA75LNGXQ	Fusion, atlas and axis combined anterior and posterior approach with combined sources of tissue using device NEC
Disc surgery	1SA75LNKDA	Fusion, atlas and axis combined anterior and posterior approach with autograft using wire, staple, button, cabling, hook
Disc surgery	1SA75LNKDK	Fusion, atlas and axis combined anterior and posterior approach with homograft [e.g. from bone bank] using wire, staple, button, cabling, hook
Disc surgery	1SA75LNKDN	Fusion, atlas and axis combined anterior and posterior approach with synthetic tissue [e.g. bone cement, paste, bioglass] using wire, staple, button, cabling, hook
Disc surgery	1SA75LNKDQ	Fusion, atlas and axis combined anterior and posterior approach with combined sources of tissue using wire, staple, button, cabling, hook
Disc surgery	1SA75LNNWA	Fusion, atlas and axis combined anterior and posterior approach with autograft using screw, screw with plate, staple or pin [e.g. odontoid screw]
Disc surgery	1SA75LNNWK	Fusion, atlas and axis combined anterior and posterior approach with homograft [e.g. from bone bank] using screw, screw with plate, staple or pin [e.g. odontoid screw]
Disc surgery	1SA75LNNWN	Fusion, atlas and axis combined anterior and posterior approach with synthetic tissue [e.g. bone cement, paste, bioglass] using screw, screw with plate, staple or pin [e.g. odontoid screw]
Disc surgery	1SA75LNNWQ	Fusion, atlas and axis combined anterior and posterior approach with combined sources of tissue using screw, screw with plate, staple or pin [e.g. odontoid screw]
Disc surgery	1SA75PFGXA	Fusion, atlas and axis posterior approach [posterolateral approach] with autograft using device NEC
Disc surgery	1SA75PFGXK	Fusion, atlas and axis posterior approach [posterolateral approach] with homograft [e.g. from bone bank] using device NEC

Disc surgery	1SA75PFGXN	Fusion, atlas and axis posterior approach [posterolateral approach] with synthetic tissue [e.g. bone cement, paste, bioglass] using device NEC
Disc surgery	1SA75PFGXQ	Fusion, atlas and axis posterior approach [posterolateral approach] with combined sources of tissue using device NEC
Disc surgery	1SA75PFKDA	Fusion, atlas and axis posterior approach [posterolateral approach] with autograft using wire, staple, button, cabling, hook
Disc surgery	1SA75PFKDK	Fusion, atlas and axis posterior approach [posterolateral approach] with homograft [e.g. from bone bank] using wire, staple, button, cabling, hook
Disc surgery	1SA75PFKDN	Fusion, atlas and axis posterior approach [posterolateral approach] with synthetic tissue [e.g. bone cement, paste, bioglass] using wire, staple, button, cabling, hook
Disc surgery	1SA75PFKDQ	Fusion, atlas and axis posterior approach [posterolateral approach] with combined sources of tissue using wire, staple, button, cabling, hook
Disc surgery	1SA75PFNWA	Fusion, atlas and axis posterior approach [posterolateral approach] with autograft using screw, screw with plate or pin [e.g. odontoid screw]
Disc surgery	1SA75PFNWK	Fusion, atlas and axis posterior approach [posterolateral approach] with homograft [e.g. from bone bank] using screw, screw with plate or pin [e.g. odontoid screw]
Disc surgery	1SA75PFNWN	Fusion, atlas and axis posterior approach [posterolateral approach] with synthetic tissue [e.g. bone cement, paste, bioglass] using screw, screw with plate or pin [e.g. odontoid screw]
Disc surgery	1SA75PFNWQ	Fusion, atlas and axis posterior approach [posterolateral approach] with combined sources of tissue using screw, screw with plate or pin [e.g. odontoid screw]
Disc surgery	1SC75ERGXA	Fusion, spinal vertebrae endoscopic [thoracoscopic, laparoscopic] anterior approach with autograft using device NEC
Disc surgery	1SC75ERGXK	Fusion, spinal vertebrae endoscopic [thoracoscopic, laparoscopic] anterior approach with homograft [e.g. from bone bank] using device NEC
Disc surgery	1SC75ERGXL	Fusion, spinal vertebrae endoscopic [thoracoscopic, laparoscopic] anterior approach with xenograft (e.g. Surgibone, Keil bone) using device NEC
Disc surgery	1SC75ERGXN	Fusion, spinal vertebrae endoscopic [thoracoscopic, laparoscopic] anterior approach with synthetic tissue [e.g. bone cement, paste, bioglass] using device NEC
Disc surgery	1SC75ERGXQ	Fusion, spinal vertebrae endoscopic [thoracoscopic, laparoscopic] anterior approach with combined sources of tissue using device NEC
Disc surgery	1SC75ERKDA	Fusion, spinal vertebrae endoscopic [thoracoscopic, laparoscopic] anterior approach with autograft approach using wire, staple, button, cabling [alone]
Disc surgery	1SC75ERKDK	Fusion, spinal vertebrae endoscopic [thoracoscopic, laparoscopic] anterior approach with homograft [e.g. from bone bank] using wire, staple, button, cabling [alone]
Disc surgery	1SC75ERKDL	Fusion, spinal vertebrae endoscopic [thoracoscopic, laparoscopic] anterior approach with xenograft (e.g.

		Surgibone, Keil bone) using wire, staple, button, cabling [alone]
Disc surgery	1SC75ERKDN	Fusion, spinal vertebrae endoscopic [thoracoscopic, laparoscopic] anterior approach with synthetic tissue [e.g. bone cement, paste, bioglass] using wire, staple, button, cabling [alone]
Disc surgery	1SC75ERKDQ	Fusion, spinal vertebrae endoscopic [thoracoscopic, laparoscopic] anterior approach with combined sources of tissue using wire, staple, button, cabling [alone]
Disc surgery	1SC75ERNWA	Fusion, spinal vertebrae endoscopic [thoracoscopic, laparoscopic] anterior approach with autograft using screw, screw with plate, staple or rod [e.g. TSRH, Zielke, pedicle screw]
Disc surgery	1SC75ERNWK	Fusion, spinal vertebrae endoscopic [thoracoscopic, laparoscopic] anterior approach with homograft [e.g. from bone bank] using screw, screw with plate, staple or rod [e.g. TSRH, Zielke, pedicle screw]
Disc surgery	1SC75ERNWL	Fusion, spinal vertebrae endoscopic [thoracoscopic, laparoscopic] anterior approach with xenograft (e.g. Surgibone, Keil bone) using screw, screw with plate, staple or rod [e.g. TSRH, Zielke, pedicle screw]
Disc surgery	1SC75ERNWN	Fusion, spinal vertebrae endoscopic [thoracoscopic, laparoscopic] anterior approach with synthetic tissue [e.g. bone cement, paste, bioglass] using screw, screw with plate, staple or rod [e.g. TSRH, Zielke, pedicle screw]
Disc surgery	1SC75ERNWQ	Fusion, spinal vertebrae, endoscopic [thoracoscopic, laparoscopic] anterior approach with combined sources of tissue using screw, screw with plate, staple or rod [e.g. TSRH, Zielke, pedicle screw]
Disc surgery	1SC75ERXXA	Fusion, spinal vertebrae endoscopic [thoracoscopic, laparoscopic] anterior approach with autograft using no device for fusion
Disc surgery	1SC75ERXXK	Fusion, spinal vertebrae endoscopic [thoracoscopic, laparoscopic] anterior approach with homograft [e.g. from bone bank] using no device for fusion
Disc surgery	1SC75ERXXL	Fusion, spinal vertebrae endoscopic [thoracoscopic, laparoscopic] anterior approach with xenograft (e.g. Surgibone, Keil bone) using no device for fusion
Disc surgery	1SC75ERXXN	Fusion, spinal vertebrae endoscopic [thoracoscopic, laparoscopic] anterior approach with synthetic tissue [e.g. bone cement, paste, bioglass] using no device for fusion
Disc surgery	1SC75ERXXQ	Fusion, spinal vertebrae endoscopic [thoracoscopic, laparoscopic] anterior approach with combined sources of tissue using no device for fusion
Disc surgery	1SC75LLGXA	Fusion, spinal vertebrae open anterior approach [anterolateral and transoral approaches] with autograft using device NEC
Disc surgery	1SC75LLGXK	Fusion, spinal vertebrae open anterior approach [anterolateral and transoral approaches] with homograft [e.g. from bone bank] using device NEC
Disc surgery	1SC75LLGXL	Fusion, spinal vertebrae open anterior approach [anterolateral and transoral approaches] with xenograft (e.g. Surgibone, Keil bone) using device NEC
Disc surgery	1SC75LLGXN	Fusion, spinal vertebrae open anterior approach [anterolateral and transoral approaches] with synthetic

		tissue [e.g. bone cement, paste, bioglass] using device NEC
Disc surgery	1SC75LLGXQ	Fusion, spinal vertebrae open anterior approach [anterolateral and transoral approaches] with combined sources of tissue using device NEC
Disc surgery	1SC75LLKDA	Fusion, spinal vertebrae open anterior approach [anterolateral and transoral approaches] with autograft using wire, staple, button, cabling (alone)
Disc surgery	1SC75LLKDK	Fusion, spinal vertebrae open anterior approach [anterolateral and transoral approaches] with homograft [e.g. from bone bank] using wire, staple, button, cabling (alone)
Disc surgery	1SC75LLKDL	Fusion, spinal vertebrae open anterior approach [anterolateral and transoral approaches] with xenograft (e.g. Surgibone, Keil bone) using wire, staple, button, cabling [alone]
Disc surgery	1SC75LLKDN	Fusion, spinal vertebrae open anterior approach [anterolateral and transoral approaches] with synthetic tissue [e.g. bone cement, paste, bioglass] using wire, staple, button, cabling (alone)
Disc surgery	1SC75LLKDQ	Fusion, spinal vertebrae open anterior approach [anterolateral and transoral approaches] with combined sources of tissue using wire, staple, button, cabling (alone)
Disc surgery	1SC75LLNWA	Fusion, spinal vertebrae open anterior approach [anterolateral and transoral approaches] with autograft using screw, screw with plate, staple or rod
Disc surgery	1SC75LLNWK	Fusion, spinal vertebrae open anterior approach [anterolateral and transoral approaches] with homograft [e.g. from bone bank] using screw, screw with plate, staple or rod
Disc surgery	1SC75LLNWL	Fusion, spinal vertebrae open anterior approach [anterolateral and transoral approaches] with xenograft (e.g. Surgibone, Keil bone) using screw, screw with plate, staple or rod
Disc surgery	1SC75LLNWN	Fusion, spinal vertebrae open anterior approach [anterolateral and transoral approaches] with synthetic tissue [e.g. bone cement, paste, bioglass] using screw, screw with plate, staple or rod
Disc surgery	1SC75LLNWQ	Fusion, spinal vertebrae open anterior approach [anterolateral and transoral approaches] with combined sources of tissue using screw, screw with plate, staple or rod
Disc surgery	1SC75LLXXA	Fusion, spinal vertebrae open anterior approach [anterolateral and transoral approaches] with autograft using no device for fusion
Disc surgery	1SC75LLXXK	Fusion, spinal vertebrae open anterior approach [anterolateral and transoral approaches] with homograft [e.g. from bone bank] using no device for fusion
Disc surgery	1SC75LLXXL	Fusion, spinal vertebrae open anterior approach [anterolateral and transoral approaches] with xenograft (e.g. Surgibone, Keil bone) using no device for fusion
Disc surgery	1SC75LLXXN	Fusion, spinal vertebrae open anterior approach [anterolateral and transoral approaches] with synthetic

		tissue [e.g. bone cement, paste, bioglass] using no device for fusion
Disc surgery	1SC75LLXXQ	Fusion, spinal vertebrae open anterior approach [anterolateral and transoral approaches] with combined sources of tissue using no device for fusion
Disc surgery	1SC75LNGXA	Fusion, spinal vertebrae open combined anterior and posterior approach [e.g. with hemi-epiphysiodesis] with autograft using device NEC
Disc surgery	1SC75LNGXK	Fusion, spinal vertebrae open combined anterior and posterior approach [e.g. with hemi-epiphysiodesis] with homograft [e.g. from bone bank] using device NEC
Disc surgery	1SC75LNGXL	Fusion, spinal vertebrae open combined anterior approach and posterior approach [e.g. with hemiepiphysiodesis] with xenograft (e.g. Surgibone, Keil bone) using device NEC
Disc surgery	1SC75LNGXN	Fusion, spinal vertebrae open combined anterior and posterior approach [e.g. with hemi-epiphysiodesis] with synthetic tissue [e.g. bone cement, paste, bioglass] using device NEC
Disc surgery	1SC75LNGXQ	Fusion, spinal vertebrae open combined anterior and posterior approach [e.g. with hemi-epiphysiodesis] with combined sources of tissue using device NEC
Disc surgery	1SC75LNKDA	Fusion, spinal vertebrae open combined anterior and posterior approach [e.g. with hemi-epiphysiodesis] with autograft using wire, staple, button, cabling (alone)
Disc surgery	1SC75LNKDK	Fusion, spinal vertebrae open combined anterior and posterior approach [e.g. with hemi-epiphysiodesis] with homograft [e.g. from bone bank] using wire, staple, button, cabling (alone)
Disc surgery	1SC75LNKDL	Fusion, spinal vertebrae open combined anterior and posterior approach [e.g. with hemiepiphysiodesis] with xenograft (e.g. Surgibone, Keil bone) using wire, staple, button, cabling [alone]
Disc surgery	1SC75LNKDN	Fusion, spinal vertebrae open combined anterior and posterior approach [e.g. with hemi-epiphysiodesis] with synthetic tissue [e.g. bone cement, paste, bioglass] using wire, staple, button, cabling (alone)
Disc surgery	1SC75LNKDQ	Fusion, spinal vertebrae open combined anterior and posterior approach [e.g. with hemi-epiphysiodesis] with combined sources of tissue using wire, staple, button, cabling (alone)
Disc surgery	1SC75LNNWA	Fusion, spinal vertebrae open combined anterior and posterior approach [e.g. with hemi-epiphysiodesis] with autograft using screw, screw with plate or rod (e.g. TSRH, Zielke, pedicle screw)
Disc surgery	1SC75LNNWK	Fusion, spinal vertebrae open combined anterior and posterior approach [e.g. with hemi-epiphysiodesis] with homograft [e.g. from bone bank] using screw, screw with plate or rod (e.g. TSRH, Zielke, pedicle screw)
Disc surgery	1SC75LNNWL	Fusion, spinal vertebrae open combined anterior and posterior approach [e.g. with hemiepiphysiodesis] with xenograft (e.g. Surgibone, Keil bone) using screw, screw with plate, staple or rod [e.g. TSRH, Zielke, pedicle screw]
Disc surgery	1SC75LNNWN	Fusion, spinal vertebrae open combined anterior and posterior approach [e.g. with hemi-epiphysiodesis] with

		synthetic tissue [e.g. bone cement, paste, bioglass] using screw, screw with plate or rod (e.g. TSRH, Zielke, pedicle screw)
Disc surgery	1SC75LNNWQ	Fusion, spinal vertebrae open combined anterior and posterior approach [e.g. with hemi-epiphysiodesis] with combined sources of tissue using screw, screw with plate or rod (e.g. TSRH, Zielke, pedicle screw)
Disc surgery	1SC75LNTCA	Fusion, spinal vertebrae open combined anterior and posterior approach [e.g. with hemi-epiphysiodesis] with autograft using rod with hook or wire [e.g. Harrington rod]
Disc surgery	1SC75LNTCK	Fusion, spinal vertebrae open combined anterior and posterior approach [e.g. with hemi-epiphysiodesis] with homograft [e.g. from bone bank] using rod with hook or wire [e.g. Harrington rod]
Disc surgery	1SC75LNTCL	Fusion, spinal vertebrae open combined anterior and posterior approach [e.g. with hemiepiphysiodesis] with xenograft (e.g. Surgibone, Keil bone) using rod with hook or wire [e.g. Harrington rod]
Disc surgery	1SC75LNTCN	Fusion, spinal vertebrae open combined anterior and posterior approach [e.g. with hemi-epiphysiodesis] with synthetic tissue [e.g. bone cement, paste, bioglass] using rod with hook or wire [e.g. Harrington rod]
Disc surgery	1SC75LNTCQ	Fusion, spinal vertebrae open combined anterior and posterior approach [e.g. with hemi-epiphysiodesis] with combined sources of tissue using rod with hook or wire [e.g. Harrington rod]
Disc surgery	1SC75PFGXA	Fusion, spinal vertebrae open posterior approach [posterolateral approach] with autograft using device NEC
Disc surgery	1SC75PFGXK	Fusion, spinal vertebrae open posterior approach [posterolateral approach] with homograft [e.g. from bone bank] using device NEC
Disc surgery	1SC75PFGXL	Fusion, spinal vertebrae open posterior approach [posterolateral approach] with xenograft (e.g. Surgibone, Keil bone) using device NEC
Disc surgery	1SC75PFGXN	Fusion, spinal vertebrae open posterior approach [posterolateral approach] with synthetic tissue [e.g. bone cement, paste, bioglass] using device NEC
Disc surgery	1SC75PFGXQ	Fusion, spinal vertebrae open posterior approach [posterolateral approach] with combined sources of tissue using device NEC
Disc surgery	1SC75PFKDA	Fusion, spinal vertebrae open posterior approach [posterolateral approach] with autograft using wire, staple, button, cabling (alone)
Disc surgery	1SC75PFKDK	Fusion, spinal vertebrae open posterior approach [posterolateral approach] with homograft [e.g. from bone bank] using wire, staple, button, cabling (alone)
Disc surgery	1SC75PFKDL	Fusion, spinal vertebrae, open posterior approach [posterolateral approach] with xenograft (e.g. Surgibone, Keil bone) using wire, staple, button, cabling [alone]
Disc surgery	1SC75PFKDN	Fusion, spinal vertebrae open posterior approach [posterolateral approach] with synthetic tissue [e.g. bone cement, paste, bioglass] using wire, staple, button, cabling (alone)

Disc surgery	1SC75PFKDQ	Fusion, spinal vertebrae open posterior approach [posterolateral approach] with combined sources of tissue using wire, staple, button, cabling (alone)
Disc surgery	1SC75PFNWA	Fusion, spinal vertebrae open posterior approach [posterolateral approach] with autograft using screw, screw with plate or rod (e.g. TSRH, Zielke, pedicle screw)
Disc surgery	1SC75PFNWK	Fusion, spinal vertebrae open posterior approach [posterolateral approach] with homograft [e.g. from bone bank] using screw, screw with plate or rod (e.g. TSRH, Zielke, pedicle screw)
Disc surgery	1SC75PFNWL	Fusion, spinal vertebrae open posterior approach [posterolateral approach] with xenograft (e.g. Surgibone, Keil bone) using screw, screw with plate, staple or rod [e.g. TSRH, Zielke, pedicle screw]
Disc surgery	1SC75PFNWN	Fusion, spinal vertebrae open posterior approach [posterolateral approach] with synthetic tissue [e.g. bone cement, paste, bioglass] using screw, screw with plate or rod (e.g. TSRH, Zielke, pedicle screw)
Disc surgery	1SC75PFNWQ	Fusion, spinal vertebrae open posterior approach [posterolateral approach] with combined sources of tissue using screw, screw with plate or rod (e.g. TSRH, Zielke, pedicle screw)
Disc surgery	1SC75PFTCA	Fusion, spinal vertebrae open posterior approach [posterolateral approach] with autograft using rod with hook or wire [e.g. Harrington rod]
Disc surgery	1SC75PFTCK	Fusion, spinal vertebrae open posterior approach [posterolateral approach] with homograft [e.g. from bone bank] using rod with hook or wire [e.g. Harrington rod]
Disc surgery	1SC75PFTCL	Fusion, spinal vertebrae open posterior approach [posterolateral approach] with xenograft (e.g. Surgibone, Keil bone) using rod with hook or wire [e.g. Harrington rod]
Disc surgery	1SC75PFTCN	Fusion, spinal vertebrae open posterior approach [posterolateral approach] with synthetic tissue [e.g. bone cement, paste, bioglass] using rod with hook or wire [e.g. Harrington rod]
Disc surgery	1SC75PFTCQ	Fusion, spinal vertebrae open posterior approach [posterolateral approach] with combined sources of tissue using rod with hook or wire [e.g. Harrington rod]
Disc surgery	1SC75PFXXA	Fusion, spinal vertebrae open posterior approach [posterolateral approach] with autograft using no device for fusion
Disc surgery	1SC75PFXXK	Fusion, spinal vertebrae open posterior approach [posterolateral approach] with homograft [e.g. from bone bank] using no device for fusion
Disc surgery	1SC75PFXXN	Fusion, spinal vertebrae open posterior approach [posterolateral approach] with synthetic tissue [e.g. bone cement, paste, bioglass] using no device for fusion
Disc surgery	1SC75PFXXQ	Fusion, spinal vertebrae open posterior approach [posterolateral approach] with combined sources of tissue using no device for fusion
Disc surgery	1SE53DAFE	Implantation of internal device, intervertebral disc using prosthetic disc [PDN] endoscopic approach [e.g. thoracoscopic, laparoscopic]

Disc surgery	1SE53DASL	Implantation of internal device, intervertebral disc using spacer device alone endoscopic approach [e.g. thoracoscopic, laparoscopic]
Disc surgery	1SE53DASLA	Implantation of internal device, intervertebral disc using spacer device and bone autograft endoscopic approach [e.g. thoracoscopic, laparoscopic]
Disc surgery	1SE53DASLK	Implantation of internal device, intervertebral disc using spacer device and bone homograft endoscopic approach [e.g. thoracoscopic, laparoscopic]
Disc surgery	1SE53DASLN	Implantation of internal device, intervertebral disc using spacer device and synthetic tissue [e.g. cement, paste] endoscopic approach [e.g. thoracoscopic, laparoscopic]
Disc surgery	1SE53DASLQ	Implantation of internal device, intervertebral disc using spacer device and bone graft with cement or paste endoscopic approach [e.g. thoracoscopic, laparoscopic]
Disc surgery	1SE53LLSL	Implantation of internal device, intervertebral disc using spacer device alone open anterior approach [anterolateral, thoracic]
Disc surgery	1SE53LLSLA	Implantation of internal device, intervertebral disc using spacer device and bone autograft open anterior approach [anterolateral, thoracic]
Disc surgery	1SE53LLSLK	Implantation of internal device, intervertebral disc using spacer device and bone homograft open anterior approach [anterolateral, thoracic]
Disc surgery	1SE53LLSLN	Implantation of internal device, intervertebral disc using spacer device and synthetic tissue [e.g. cement, paste] open anterior approach [anterolateral, thoracic]
Disc surgery	1SE53LLSLQ	Implantation of internal device, intervertebral disc using spacer device and bone graft with cement or paste open anterior approach [anterolateral, thoracic]
Disc surgery	1SE53PFFE	Implantation of internal device, intervertebral disc using prosthetic disc [PDN] open posterior approach [posterolateral]
Disc surgery	1SE53PFSL	Implantation of internal device, intervertebral disc using spacer device alone open posterior approach [posterolateral]
Disc surgery	1SE53PFSLA	Implantation of internal device, intervertebral disc using spacer device and bone autograft open posterior approach [posterolateral]
Disc surgery	1SE53PFSLK	Implantation of internal device, intervertebral disc using spacer device and bone homograft open posterior approach [posterolateral]
Disc surgery	1SE53PFSLN	Implantation of internal device, intervertebral disc using spacer device and synthetic tissue [e.g. cement, paste] open posterior approach [posterolateral]
Disc surgery	1SE53PFSLQ	Implantation of internal device, intervertebral disc using spacer device and bone graft with cement or paste open posterior approach [posterolateral]
Disc surgery	1SE59HAAW	Destruction, intervertebral disc using radiofrequency probe and percutaneous approach
Disc surgery	1SE59HACG	Destruction, intervertebral disc using microwave device and percutaneous approach
Disc surgery	1SE59HAKK	Destruction, intervertebral disc using electrical heat device (for electrothermy) and percutaneous approach

Disc surgery	1SE59HAX7	Destruction, intervertebral disc using chemical agent and percutaneous approach
Disc surgery	1SE59LAAG	Destruction, intervertebral disc using laser and open approach
Disc surgery	1SE59LAAW	Destruction, intervertebral disc using radiofrequency probe and open approach
Disc surgery	1SE59LAGX	Destruction, intervertebral disc using device NEC and open approach
Disc surgery	1SE87DA	Excision partial, intervertebral disc endoscopic approach without tissue
Disc surgery	1SE87HA	Excision partial, intervertebral disc percutaneous approach without tissue
Disc surgery	1SE87LL	Excision partial, intervertebral disc open anterior [anterolateral] approach without tissue
Disc surgery	1SE87LLXXA	Excision partial, intervertebral disc open anterior [anterolateral] approach using bone autograft
Disc surgery	1SE87LLXXK	Excision partial, intervertebral disc open anterior [anterolateral] approach using bone homograft
Disc surgery	1SE87LLXXL	Excision partial, intervertebral disc open anterior [anterolateral] approach using bone xenograft [e.g. bovine bone]
Disc surgery	1SE87PF	Excision partial, intervertebral disc open posterior [posterolateral] approach without tissue
Disc surgery	1SE87PFXXA	Excision partial, intervertebral disc open posterior [posterolateral] approach using bone autograft
Disc surgery	1SE87PFXXK	Excision partial, intervertebral disc open posterior [posterolateral] approach using bone homograft
Disc surgery	1SE87PFXXL	Excision partial, intervertebral disc open posterior [posterolateral] approach using bone xenograft [e.g. bovine bone]
Eardrum and/or mastoid repair	1DF80XG	Repair, tympanic membrane without tissue tympanoplasty, type 1
Eardrum and/or mastoid repair	1DF80XGXXA	Repair, tympanic membrane using full thickness autograft [e.g. fascia], tympanoplasty type 1
Eardrum and/or mastoid repair	1DF80XGXXN	Repair, tympanic membrane using synthetic material, [e.g. ceramic, metal] tympanoplasty type 1
Eardrum and/or mastoid repair	1DF80XGXXQ	Repair, tympanic membrane using combined tissue types (e.g. full/split grafts, synthetic material) tympanoplasty type 1
Eardrum and/or mastoid repair	1DF80XH	Repair, tympanic membrane without tissue tympanoplasty type 2 (that with ossicular repair, stapes is present)
Eardrum and/or mastoid repair	1DF80XHXXA	Repair, tympanic membrane using full thickness autograft [e.g. fascia], tympanoplasty type 2 (that with ossicular repair, stapes is present)
Eardrum and/or mastoid repair	1DF80XHXXK	Repair, tympanic membrane using homograft [e.g. deceased donor ossicles], tympanoplasty type 2 (that with ossicular repair, stapes is present)
Eardrum and/or mastoid repair	1DF80XHXXL	Repair, tympanic membrane using xenograft, tympanoplasty type 2 (that with ossicular repair, stapes is present)
Eardrum and/or mastoid repair	1DF80XHXXN	Repair, tympanic membrane using synthetic material [e.g. ceramic, metal] tympanoplasty type 2 (that with ossicular repair, stapes is present)

Eardrum and/or mastoid repair	1DF80XHXXQ	Repair, tympanic membrane using combined tissue types (e.g. full/split grafts, synthetic material) tympanoplasty type 2 (that with ossicular repair, stapes is present)
Eardrum and/or mastoid repair	1DF80XJXXA	Repair, tympanic membrane using full thickness autograft [e.g. fascia] tympanoplasty type 3 (that with ossicular repair, stapes is absent or severely defective)
Eardrum and/or mastoid repair	1DF80XJXXK	Repair, tympanic membrane using homograft [e.g. deceased donor ossicles], tympanoplasty type 3 (that with ossicular repair, stapes is absent or severely defective)
Eardrum and/or mastoid repair	1DF80XJXXN	Repair, tympanic membrane using synthetic material [e.g. ceramic, metal], tympanoplasty type 3 (that with ossicular repair, stapes is absent or severely defective)
Eardrum and/or mastoid repair	1DF80XJXXQ	Repair, tympanic membrane using combined tissue types (e.g. full/split grafts, synthetic material) tympanoplasty type 3 (that with ossicular repair, stapes is absent or severely defective)
Eardrum and/or mastoid repair	1DF80XKXXA	Repair, tympanic membrane using full thickness autograft [e.g. fascia], tympanoplasty type 4
Eardrum and/or mastoid repair	1DF80XKXXQ	Repair, tympanic membrane using combined tissue types [e.g. full/split grafts, synthetic material], tympanoplasty type 4
Eardrum and/or mastoid repair	1DF80XL	Repair, tympanic membrane without tissue, tympanoplasty type 5
Eardrum and/or mastoid repair	1DF80XLXXA	Repair, tympanic membrane using full thickness autograft [e.g. fascia], tympanoplasty type 5
Eardrum and/or mastoid repair	1DF80XLXXQ	Repair, tympanic membrane using combined tissue types [e.g. full/split grafts, synthetic material] tympanoplasty type 5
Eardrum and/or mastoid repair	1DL80LAXXA	Repair, mastoid (process) open approach using autograft [e.g. fascia, cartilage, bone]
Eardrum and/or mastoid repair	1DL80LAXXF	Repair, mastoid (process) open approach using free flap
Eardrum and/or mastoid repair	1DL80LAXXG	Repair, mastoid (process) open approach using pedicled flap [e.g. pedicled muscle flap, Rambo procedure]
Eardrum and/or mastoid repair	1DL80LAXXQ	Repair, mastoid (process) open approach using combined sources of tissue [e.g. autograft, pedicled/free flap]
Eardrum and/or mastoid repair	1DL80SZXXA	Repair, mastoid (process) open craniotomy/craniectomy [temporal] approach using autograft [e.g. fascia, cartilage, bone]
Eardrum and/or mastoid repair	1DL80SZXXF	Repair, mastoid (process) open craniotomy/craniectomy [temporal] approach using free flap
Eardrum and/or mastoid repair	1DL80SZXXG	Repair, mastoid (process) open craniotomy/craniectomy [temporal] approach using pedicled flap [e.g. pedicled muscle flap, Rambo procedure]
Eardrum and/or mastoid repair	1DL80SZXXQ	Repair, mastoid (process) open craniotomy/craniectomy [temporal] approach using combined sources of tissue [e.g. autograft, pedicled/free flap]
Fractures	1EA73LA	Reduction, cranium using open approach
Fractures	1EA74LAKD	Fixation, cranium no tissue used during fixation using wire or mesh only
Fractures	1EA74LAKDA	Fixation, cranium with autograft using wire or mesh only
Fractures	1EA74LAKDF	Fixation, cranium with free flap using wire or mesh only
Fractures	1EA74LAKDG	Fixation, cranium with pedicled flap [pericranial flap] using wire or mesh only

Fractures	1EA74LAKDK	Fixation, cranium with homograft using wire or mesh only
Fractures	1EA74LAKDN	Fixation, cranium with synthetic tissue [cement, paste] using wire or mesh only
Fractures	1EA74LAKDQ	Fixation, cranium with combined sources of tissue using wire or mesh only
Fractures	1EA74LANW	Fixation, cranium no tissue used during fixation using plate, screw device (with or without wire or mesh)
Fractures	1EA74LANWA	Fixation, cranium with autograft using plate, screw device (with or without wire or mesh)
Fractures	1EA74LANWF	Fixation, cranium with free flap using plate, screw device (with or without wire or mesh)
Fractures	1EA74LANWG	Fixation, cranium with pedicled flap [pericranial flap] using plate, screw device (with or without wire or mesh)
Fractures	1EA74LANWK	Fixation, cranium with homograft using plate, screw device (with or without wire or mesh)
Fractures	1EA74LANWN	Fixation, cranium with synthetic tissue[cement, paste] using plate, screw device (with or without wire or mesh)
Fractures	1EA74LANWQ	Fixation, cranium with combined sources of tissue using plate, screw device (with or without wire or mesh)
Fractures	1EB73JA	Reduction, zygoma using external (closed) approach
Fractures	1EB73LA	Reduction, zygoma using open approach
Fractures	1EB74LAKD	Fixation, zygoma without tissue [device only] using wire or mesh only
Fractures	1EB74LAKDA	Fixation, zygoma with autograft using wire or mesh only
Fractures	1EB74LAKDN	Fixation, zygoma with synthetic tissue[cement, paste] using wire or mesh only
Fractures	1EB74LAKDQ	Fixation, zygoma with combined sources of tissue using wire or mesh only
Fractures	1EB74LANW	Fixation, zygoma without tissue [device only] using plate, screw device (with or without wire or mesh)
Fractures	1EB74LANWA	Fixation, zygoma with autograft using plate, screw device (with or without wire or mesh)
Fractures	1EB74LANWN	Fixation, zygoma with synthetic tissue[cement, paste] using plate, screw device (with or without wire or mesh)
Fractures	1EB74LANWQ	Fixation, zygoma with combined sources of tissue using plate, screw device (with or without wire or mesh)
Fractures	1EC74LAKD	Fixation, nasoethmoid and orbital complex without tissue [device only] using wire or mesh only
Fractures	1EC74LAKDA	Fixation, nasoethmoid and orbital complex with autograft using wire or mesh only
Fractures	1EC74LAKDF	Fixation, nasoethmoid and orbital complex with free flap using wire or mesh only
Fractures	1EC74LAKDG	Fixation, nasoethmoid and orbital complex with pedicled flap [pericranial flap] using wire or mesh only
Fractures	1EC74LAKDK	Fixation, nasoethmoid and orbital complex with homograft using wire or mesh only
Fractures	1EC74LAKDN	Fixation, nasoethmoid and orbital complex with synthetic tissue [cement, paste] using wire or mesh only
Fractures	1EC74LAKDQ	Fixation, nasoethmoid and orbital complex with combined sources of tissue using wire or mesh only
Fractures	1EC74LANW	Fixation, nasoethmoid and orbital complex without tissue [device only] using plate, screw device (with/without wire/mesh)

Fractures	1EC74LANWA	Fixation, nasoethmoid and orbital complex with autograft using plate, screw device (with/without wire/mesh)
Fractures	1EC74LANWF	Fixation, nasoethmoid and orbital complex with free flap using plate, screw device (with/without wire/mesh)
Fractures	1EC74LANWG	Fixation, nasoethmoid and orbital complex with pedicled flap [pericranial flap] using plate, screw device (with/without wire/mesh)
Fractures	1EC74LANWK	Fixation, nasoethmoid and orbital complex with homograft using plate, screw device (with/without wire/mesh)
Fractures	1EC74LANWN	Fixation, nasoethmoid and orbital complex with synthetic tissue [cement, paste] using plate, screw device (with/without wire/mesh)
Fractures	1EC74LANWQ	Fixation, nasoethmoid and orbital complex with combined sources of tissue using plate, screw device (with/without wire/mesh)
Fractures	1ED73JA	Reduction, maxilla using closed (external) approach
Fractures	1ED74LAKD	Fixation, maxilla no tissue used [device only] using wire or mesh only
Fractures	1ED74LAKDA	Fixation, maxilla with autograft using wire or mesh only
Fractures	1ED74LAKDF	Fixation, maxilla with free flap using wire or mesh only
Fractures	1ED74LAKDG	Fixation, maxilla with pedicled flap using wire or mesh only
Fractures	1ED74LAKDK	Fixation, maxilla with homograft using wire or mesh only
Fractures	1ED74LAKDN	Fixation, maxilla with synthetic tissue[cement, paste] using wire or mesh only
Fractures	1ED74LAKDQ	Fixation, maxilla with combined sources of tissue using wire or mesh only
Fractures	1ED74LANW	Fixation, maxilla no tissue used [device only] using plate, screw device (with/without wire/mesh)
Fractures	1ED74LANWA	Fixation, maxilla with autograft using plate, screw device (with/without wire/mesh)
Fractures	1ED74LANWF	Fixation, maxilla with free flap using plate, screw device (with/without wire/mesh)
Fractures	1ED74LANWG	Fixation, maxilla with pedicled flap using plate, screw device (with/without wire/mesh)
Fractures	1ED74LANWK	Fixation, maxilla with homograft using plate, screw device (with/without wire/mesh)
Fractures	1ED74LANWN	Fixation, maxilla with synthetic tissue[cement, paste] using plate, screw device (with/without wire/mesh)
Fractures	1ED74LANWQ	Fixation, maxilla with combined sources of tissue using plate, screw device (with/without wire/mesh)
Fractures	1EE03HAKC	Immobilization, mandible using percutaneous external fixator [e.g. biphase device]
Fractures	1EE73JA	Reduction, mandible using closed [external] approach
Fractures	1EE74LAKD	Fixation, open approach mandible without tissue [device only] using wire or mesh only
Fractures	1EE74LAKDA	Fixation, mandible open approach with autograft using wire or mesh only
Fractures	1EE74LAKDF	Fixation, mandible open approach with free flap [fibular or costochondral flap] using wire or mesh only
Fractures	1EE74LAKDK	Fixation, mandible open approach with homograft using wire or mesh only

Fractures	1EE74LAKDN	Fixation, mandible open approach with synthetic tissue[cement, paste] using wire or mesh only
Fractures	1EE74LAKDQ	Fixation, mandible open approach with combined sources of tissue using wire or mesh only
Fractures	1EE74LANW	Fixation, mandible open approach without tissue [device only] using plate, screw device (with/without wire/mesh)
Fractures	1EE74LANWA	Fixation, mandible open approach with autograft using plate, screw device (with/without wire/mesh)
Fractures	1EE74LANWF	Fixation, mandible open approach with free flap [fibular or costochondral flap] using plate, screw device (with/without wire/mesh)
Fractures	1EE74LANWK	Fixation, mandible open approach with homograft using plate, screw device (with/without wire/mesh)
Fractures	1EE74LANWN	Fixation, mandible open approach with synthetic tissue[cement, paste] using plate, screw device (with/without wire/mesh)
Fractures	1EE74LANWQ	Fixation, mandible open approach with combined sources of tissue using plate, screw device (with/without wire/mesh)
Fractures	1EF73JA	Reduction, maxilla with mandible using closed [external] approach
Fractures	1EF74LAKD	Fixation, maxilla with mandible open approach no tissue used [device only] using wire or mesh only
Fractures	1EF74LAKDA	Fixation, maxilla with mandible open approach with autograft using wire or mesh only
Fractures	1EF74LAKDF	Fixation, maxilla with mandible open approach with free [distant] flap using wire or mesh only
Fractures	1EF74LAKDK	Fixation, maxilla with mandible open approach with homograft using wire or mesh only
Fractures	1EF74LAKDN	Fixation, maxilla with mandible open approach with synthetic tissue [cement, paste] using wire or mesh only
Fractures	1EF74LAKDQ	Fixation, maxilla with mandible open approach with combined sources of tissue using wire or mesh only
Fractures	1EF74LANW	Fixation, maxilla with mandible open approach no tissue used [device only] using plate, screw device (with/without wire/mesh)
Fractures	1EF74LANWA	Fixation, maxilla with mandible open approach with autograft using plate, screw device (with/without wire/mesh)
Fractures	1EF74LANWF	Fixation, maxilla with mandible open approach with free [distant] flap using plate, screw device (with/without wire/mesh)
Fractures	1EF74LANWK	Fixation, maxilla with mandible open approach with homograft using plate, screw device (with/without wire/mesh)
Fractures	1EF74LANWN	Fixation, maxilla with mandible open approach with synthetic tissue [cement, paste] using plate, screw device (with/without wire/mesh)
Fractures	1EF74LANWQ	Fixation, maxilla with mandible open approach with combined sources of tissue using plate, screw device (with/without wire/mesh)
Fractures	1EG74LAKD	Fixation, multiple bones of mid face region, without cranium involvement no tissue used [device only] using wire or mesh only

Fractures	1EG74LAKDA	Fixation, multiple bones of mid face region, without cranium involvement with autograft using wire or mesh only
Fractures	1EG74LAKDF	Fixation, multiple bones of mid face region, without cranium involvement with free flap using wire or mesh only
Fractures	1EG74LAKDG	Fixation, multiple bones of mid face region, without cranium involvement with pedicled flap [cranial flap] using wire or mesh only
Fractures	1EG74LAKDK	Fixation, multiple bones of mid face region, without cranium involvement with homograft using wire or mesh only
Fractures	1EG74LAKDN	Fixation, multiple bones of mid face region, without cranium involvement with synthetic tissue [cement, paste] using wire or mesh only
Fractures	1EG74LAKDQ	Fixation, multiple bones of mid face region, without cranium involvement with combined sources of tissue using wire or mesh only
Fractures	1EG74LANW	Fixation, multiple bones of mid face region, without cranium involvement no tissue used [device only] using plate, screw device (with/without wire/mesh)
Fractures	1EG74LANWA	Fixation, multiple bones of mid face region, without cranium involvement with autograft using plate, screw device (with/without wire/mesh)
Fractures	1EG74LANWF	Fixation, multiple bones of mid face region, without cranium involvement with free flap using plate, screw device (with/without wire/mesh)
Fractures	1EG74LANWG	Fixation, multiple bones of mid face region, without cranium involvement with pedicled flap [cranial flap] using plate, screw device (with/without wire/mesh)
Fractures	1EG74LANWK	Fixation, multiple bones of mid face region, without cranium involvement with homograft using plate, screw device (with/without wire/mesh)
Fractures	1EG74LANWN	Fixation, multiple bones of mid face region, without cranium involvement with synthetic tissue [cement, paste] using plate, screw device (with/without wire/mesh)
Fractures	1EG74LANWQ	Fixation, multiple bones of mid face region, without cranium involvement with combined sources of tissue using plate, screw device (with/without wire/mesh)
Fractures	1EH74LAKD	Fixation, multiple bones of mid face region, with cranium involvement no tissue used [device only] using wire or mesh only
Fractures	1EH74LAKDA	Fixation, multiple bones of mid face region, with cranium involvement with autograft using wire or mesh only
Fractures	1EH74LAKDF	Fixation, multiple bones of mid face region, with cranium involvement with free flap using wire or mesh only
Fractures	1EH74LAKDG	Fixation, multiple bones of mid face region, with cranium involvement with pedicled flap using wire or mesh only
Fractures	1EH74LAKDK	Fixation, multiple bones of mid face region, with cranium involvement with homograft using wire or mesh only
Fractures	1EH74LAKDN	Fixation, multiple bones of mid face region, with cranium involvement with synthetic tissue [cement, paste] using wire or mesh only

Fractures	1EH74LAKDQ	Fixation, multiple bones of mid face region, with cranium involvement with combined sources of tissue using wire or mesh only
Fractures	1EH74LANW	Fixation, multiple bones of mid face region, with cranium involvement no tissue used [device only] using plate, screw device (with/without wire/mesh)
Fractures	1EH74LANWA	Fixation, multiple bones of mid face region, with cranium involvement with autograft using plate, screw device (with/without wire/mesh)
Fractures	1EH74LANWF	Fixation, multiple bones of mid face region, with cranium involvement with free flap using plate, screw device (with/without wire/mesh)
Fractures	1EH74LANWG	Fixation, multiple bones of mid face region, with cranium involvement with pedicled flap using plate, screw device (with/without wire/mesh)
Fractures	1EH74LANWK	Fixation, multiple bones of mid face region, with cranium involvement with homograft using plate, screw device (with/without wire/mesh)
Fractures	1EH74LANWN	Fixation, multiple bones of mid face region, with cranium involvement with synthetic tissue [cement, paste] using plate, screw device (with/without wire/mesh)
Fractures	1EH74LANWQ	Fixation, multiple bones of mid face region, with cranium involvement with combined sources of tissue using plate, screw device (with/without wire/mesh)
Fractures	1EL74LAKDK	Fixation, temporomandibular joint [TMJ] open approach with homograft using wire or mesh device only
Fractures	1EL74LANWK	Fixation, temporomandibular joint [TMJ] open approach with homograft using plate, screw device (with or without wire or mesh)
Fractures	1ET73LA	Reduction, nose using open approach
Fractures	1SA03HAKC	Immobilization, atlas and axis immobilization alone with percutaneous fixator device [tongs, halo]
Fractures	1SA03JZKC	Immobilization, atlas and axis immobilization with traction or traction alone with percutaneous fixator device [tongs, halo]
Fractures	1SA74LLGX	Fixation, atlas and axis open anterior approach [Includes: anterolateral, transoral and retropharyngeal approaches] using device NEC
Fractures	1SA74LLKD	Fixation, atlas and axis open anterior approach [Includes: anterolateral, transoral and retropharyngeal approaches] using wire, staple, button, cabling, hook
Fractures	1SA74LLNW	Fixation, atlas and axis open anterior approach [Includes: anterolateral, transoral and retropharyngeal approaches] using screw, screw with plate, staple or pin [e.g. odontoid screw]
Fractures	1SA74LNGX	Fixation, atlas and axis combined open anterior with posterior approach using device NEC
Fractures	1SA74LNKD	Fixation, atlas and axis combined open anterior with posterior approach using wire, staple, button, cabling, hook
Fractures	1SA74LNNW	Fixation, atlas and axis combined open anterior with posterior approach using screw, screw with plate, staple or pin [e.g. odontoid screw]
Fractures	1SA74PFGX	Fixation, atlas and axis open posterior approach [Includes: posterolateral approach] using device NEC

Fractures	1SA74PFKD	Fixation, atlas and axis open posterior approach [Includes: posterolateral approach] using wire, staple, button, cabling, hook
Fractures	1SA74PFNW	Fixation, atlas and axis open posterior approach [Includes: posterolateral approach] using screw, screw with plate, staple or pin [e.g. odontoid screw]
Fractures	1SC03HAKC	Immobilization, spinal vertebrae immobilization alone with percutaneous fixator device [tongs, halo]
Fractures	1SC03JZKC	Immobilization, spinal vertebrae immobilization with traction or traction alone with percutaneous fixator device [tongs, halo]
FRACTURES	1SC74HAMK	Fixation, spinal vertebrae percutaneous approach using dynamic stabilization system [e.g. Aperius, Dynesys, DIAM, Xstop, Wallis, Coflex]
Fractures	1SC74LLGX	Fixation, spinal vertebrae open anterior (anterolateral) approach using device NEC
Fractures	1SC74LLKD	Fixation, spinal vertebrae open anterior (anterolateral) approach using wire, staple, button, cabling [alone]
Fractures	1SC74LLMK	Fixation, spinal vertebrae open anterior (anterolateral) approach using dynamic stabilization system [e.g. Aperius, Dynesys, DIAM, Xstop, Wallis, Coflex]
Fractures	1SC74LLNW	Fixation, spinal vertebrae open anterior (anterolateral) approach using screw, screw with plate or rod
Fractures	1SC74LLTC	Fixation, spinal vertebrae open anterior (anterolateral) approach using rod with hook or wire [e.g. Phoenix magnetic rod]
Fractures	1SC74PFGX	Fixation, spinal vertebrae open posterior (posterolateral) approach using device NEC
Fractures	1SC74PFKD	Fixation, spinal vertebrae open posterior (posterolateral) approach using wire, staple, button, cabling [alone]
Fractures	1SC74PFMK	Fixation, spinal vertebrae open posterior (posterolateral) approach using dynamic stabilization system [e.g. Aperius, Dynesys, DIAM, Xstop, Wallis, Coflex]
Fractures	1SC74PFNW	Fixation, spinal vertebrae open posterior (posterolateral) approach using screw, screw with plate or rod
Fractures	1SC74PFTC	Fixation, spinal vertebrae open posterior (posterolateral) approach using rod with hook or wire [e.g. Phoenix magnetic rod]
Fractures	1SF73JA	Reduction, sacrum and coccyx using closed external approach
Fractures	1SF73PF	Reduction, sacrum and coccyx using posterior approach
Fractures	1SF74HANW	Fixation, sacrum and coccyx using percutaneous approach and screw, screw with plate
Fractures	1SF74LLNW	Fixation, sacrum and coccyx using anterior approach and screw, screw with plate/rod
Fractures	1SF74LLTC	Fixation, sacrum and coccyx using anterior approach and rod, rod with nuts/bolts
Fractures	1SF74PFNW	Fixation, sacrum and coccyx using posterior approach and screw, screw with plate
Fractures	1SI74HANW	Fixation, sacroiliac joint using percutaneous approach and screw
Fractures	1SI74PFKD	Fixation, sacroiliac joint using posterior approach and wire or staple
Fractures	1SI74PFNW	Fixation, sacroiliac joint using posterior approach and plate, screw device

Fractures	1SK73JA	Reduction, sternum using closed (external) approach
Fractures	1SK73LA	Reduction, sternum using open approach (with or without removal of bone fragments)
Fractures	1SK74LAKD	Fixation, sternum using open approach and wire/mesh/staple
Fractures	1SK74LANW	Fixation, sternum using open approach and screw/screw with plate
Fractures	1SL73JA	Reduction, ribs using closed (external) approach
Fractures	1SL73LA	Reduction, ribs using open approach (with or without removal of bony fragments)
Fractures	1SL74LLKD	Fixation, ribs using anterior approach and wire/mesh/staple
Fractures	1SL74LLNW	Fixation, ribs using anterior approach and plate, screw
Fractures	1SL74PFKD	Fixation, ribs using posterior approach and wire/mesh/staple
Fractures	1SL74PFNW	Fixation, ribs using posterior approach and plate, screw
Fractures	1SM03HAKC	Immobilization, clavicle using percutaneous external fixator
Fractures	1SM73JA	Reduction, clavicle using closed (external) approach
Fractures	1SM73LA	Reduction, clavicle using open approach (with or without removal of bony fragments)
Fractures	1SM74HAKD	Fixation, clavicle percutaneous approach with no tissue used using wire, tension band, staple or mesh
Fractures	1SM74HANW	Fixation, clavicle percutaneous approach with no tissue used using screw, screw with plate
Fractures	1SM74LAKD	Fixation, clavicle open approach with no tissue used using wire, tension band, staple or mesh
Fractures	1SM74LAKDA	Fixation, clavicle open approach with bone autograft using wire, tension band, staple or mesh
Fractures	1SM74LAKDK	Fixation, clavicle open approach with homograft (and fixative device) [e.g. allograft matrix] using wire, tension band, staple or mesh
Fractures	1SM74LAKDQ	Fixation, clavicle open approach with combined sources of tissue using wire, tension band, staple or mesh
Fractures	1SM74LALQ	Fixation, clavicle open approach with no tissue used using intramedullary nail
Fractures	1SM74LALQA	Fixation, clavicle open approach with bone autograft using intramedullary nail
Fractures	1SM74LALQK	Fixation, clavicle open approach with homograft (and fixative device) [e.g. allograft matrix] using intramedullary nail
Fractures	1SM74LALQN	Fixation, clavicle open approach with synthetic tissue [e.g. cement, paste] using intramedullary nail
Fractures	1SM74LALQQ	Fixation, clavicle open approach with combined sources of tissue using intramedullary nail
Fractures	1SM74LANV	Fixation, clavicle open approach with no tissue used using pin
Fractures	1SM74LANVA	Fixation, clavicle open approach with bone autograft using pin
Fractures	1SM74LANVK	Fixation, clavicle open approach with homograft (and fixative device) [e.g. allograft matrix] using pin
Fractures	1SM74LANVN	Fixation, clavicle open approach with synthetic tissue [e.g. cement, paste] using pin

Fractures	1SM74LANVQ	Fixation, clavicle open approach with combined sources of tissue using pin
Fractures	1SM74LANW	Fixation, clavicle open approach with no tissue used using screw, screw with plate
Fractures	1SM74LANWA	Fixation, clavicle open approach with bone autograft using screw, screw with plate
Fractures	1SM74LANWK	Fixation, clavicle open approach with homograft (and fixative device) [e.g. allograft matrix] using screw, screw with plate
Fractures	1SM74LANWN	Fixation, clavicle open approach with synthetic tissue [e.g. cement, paste] using screw, screw with plate
Fractures	1SM74LANWQ	Fixation, clavicle open approach with combined sources of tissue using screw, screw with plate
Fractures	1SN73JA	Reduction, scapula using closed (external) approach
Fractures	1SN74LAKD	Fixation, scapula using fixation device only using wire, mesh, staple
Fractures	1SN74LAKDA	Fixation, scapula using bone autograft using wire, mesh, staple
Fractures	1SN74LAKDK	Fixation, scapula using bone homograft using wire, mesh, staple
Fractures	1SN74LAKDN	Fixation, scapula using synthetic tissue [e.g. bone cement or paste] using wire, mesh, staple
Fractures	1SN74LAKDQ	Fixation, scapula using combined sources of tissue [e.g. bone graft, cement/paste] using wire, mesh, staple
Fractures	1SN74LANV	Fixation, scapula using fixation device only using pin, nail
Fractures	1SN74LANVA	Fixation, scapula using bone autograft using pin, nail
Fractures	1SN74LANVK	Fixation, scapula using bone homograft using pin, nail
Fractures	1SN74LANVN	Fixation, scapula using synthetic tissue [e.g. bone cement or paste] using pin, nail
Fractures	1SN74LANVQ	Fixation, scapula using combined sources of tissue [e.g. bone graft, cement/paste] using pin, nail
Fractures	1SN74LANW	Fixation, scapula using fixation device only using screw, plate and screw
Fractures	1SN74LANWA	Fixation, scapula using bone autograft using screw, plate and screw
Fractures	1SN74LANWK	Fixation, scapula using bone homograft using screw, plate and screw
Fractures	1SN74LANWN	Fixation, scapula using synthetic tissue [e.g. bone cement or paste] using screw, plate and screw
Fractures	1SN74LANWQ	Fixation, scapula using combined sources of tissue [e.g. bone graft, cement/paste] using screw, plate and screw
Fractures	1SQ03HAKC	Immobilization, pelvis immobilization alone with percutaneous external fixator
Fractures	1SQ73JA	Reduction, pelvis using closed (external) approach
Fractures	1SQ73LA	Reduction, pelvis using open approach
Fractures	1SQ74LAKD	Fixation, pelvis using fixation device alone using wire, staple
Fractures	1SQ74LAKDA	Fixation, pelvis using bone autograft using wire, staple
Fractures	1SQ74LAKDK	Fixation, pelvis using bone homograft using wire, staple
Fractures	1SQ74LAKDN	Fixation, pelvis using synthetic tissue [e.g. bone cement, or paste] using wire, staple

Fractures	1SQ74LAKDQ	Fixation, pelvis using combined bone graft and cement, or paste using wire, staple
Fractures	1SQ74LANV	Fixation, pelvis using fixation device alone using pin, nail
Fractures	1SQ74LANVA	Fixation, pelvis using bone autograft using pin, nail
Fractures	1SQ74LANVK	Fixation, pelvis using bone homograft using pin, nail
Fractures	1SQ74LANVN	Fixation, pelvis using synthetic tissue [e.g. bone cement, or paste] using pin, nail
Fractures	1SQ74LANVQ	Fixation, pelvis using combined bone graft and cement, or paste using pin, nail
Fractures	1SQ74LANW	Fixation, pelvis using fixation device alone using screw, screw with plate (with/without wire, pin or nails)
Fractures	1SQ74LANWA	Fixation, pelvis using bone autograft using screw, screw with plate (with/without wire, pin or nails)
Fractures	1SQ74LANWK	Fixation, pelvis using bone homograft using screw, screw with plate (with/without wire, pin or nails)
Fractures	1SQ74LANWN	Fixation, pelvis using synthetic tissue [e.g. bone cement, or paste] using screw, screw with plate (with/without wire, pin or nails)
Fractures	1SQ74LANWQ	Fixation, pelvis using combined bone graft and cement, or paste using screw, screw with plate (with/without wire, pin or nails)
Fractures	1SW73JA	Reduction, pubis using closed (external) approach
Fractures	1SW74LAKD	Fixation, pubis using open approach and wire/mesh fixation
Fractures	1SW74LANV	Fixation, pubis using open approach and pin/nail fixation
Fractures	1SW74LANW	Fixation, pubis using open approach and screw/plate fixation
Fractures	1TK03HAFQ	Immobilization, humerus with percutaneous traction[e.g. skeletal] using cast [spica, cylinder]
Fractures	1TK03HAKC	Immobilization, humerus with percutaneous traction[e.g. skeletal] using percutaneous external fixator
Fractures	1TK03HASR	Immobilization, humerus with percutaneous traction[e.g. skeletal] using splinting device
Fractures	1TK03HATA	Immobilization, humerus with percutaneous traction[e.g. skeletal] using traction alone
Fractures	1TK73JA	Reduction, humerus using closed [external] approach
Fractures	1TK73LA	Reduction, humerus using open approach
Fractures	1TK74HAKD	Fixation, humerus percutaneous approach [e.g. with closed or no reduction] fixation device alone using wire, mesh, staple
Fractures	1TK74HALQ	Fixation, humerus percutaneous approach [e.g. with closed or no reduction] fixation device alone using intramedullary nail
Fractures	1TK74HANV	Fixation, humerus percutaneous approach [e.g. with closed or no reduction] fixation device alone using pin, nail
Fractures	1TK74HANW	Fixation, humerus using percutaneous approach [e.g. with closed or no reduction] fixation device alone using plate, screw
Fractures	1TK74LA	Fixation, humerus open approach using no device used (e.g. for epiphysiodesis)
Fractures	1TK74LAKD	Fixation, humerus open approach fixation device alone using wire, mesh, staple

Fractures	1TK74LAKDA	Fixation, humerus open approach with bone autograft using wire, mesh, staple
Fractures	1TK74LAKDK	Fixation, humerus open approach with bone homograft using wire, mesh, staple
Fractures	1TK74LAKDN	Fixation, humerus open approach with synthetic tissue [e.g. bone cement or paste] using wire, mesh, staple
Fractures	1TK74LAKDQ	Fixation, humerus open approach with combined bone graft and cement, paste using wire, mesh, staple
Fractures	1TK74LALQ	Fixation, humerus open approach fixation device alone using intramedullary nail
Fractures	1TK74LALQA	Fixation, humerus open approach with bone autograft using intramedullary nail
Fractures	1TK74LALQK	Fixation, humerus open approach with bone homograft using intramedullary nail
Fractures	1TK74LALQN	Fixation, humerus open approach with synthetic tissue [e.g. bone cement or paste] using intramedullary nail
Fractures	1TK74LALQQ	Fixation, humerus open approach with combined bone graft and cement, paste using intramedullary nail
Fractures	1TK74LANV	Fixation, humerus open approach fixation device alone using pin, nail
Fractures	1TK74LANVA	Fixation, humerus open approach with bone autograft using pin, nail
Fractures	1TK74LANVK	Fixation, humerus open approach with bone homograft using pin, nail
Fractures	1TK74LANVN	Fixation, humerus open approach with synthetic tissue [e.g. bone cement or paste] using pin, nail
Fractures	1TK74LANVQ	Fixation, humerus open approach with combined bone graft and cement, paste using pin, nail
Fractures	1TK74LANW	Fixation, humerus open approach fixation device alone using plate, screw
Fractures	1TK74LANWA	Fixation, humerus open approach with bone autograft using plate, screw
Fractures	1TK74LANWK	Fixation, humerus open approach with bone homograft using plate, screw
Fractures	1TK74LANWN	Fixation, humerus open approach with synthetic tissue [e.g. bone cement or paste] using plate, screw
Fractures	1TK74LANWQ	Fixation, humerus open approach with combined bone graft and cement, paste using plate, screw
Fractures	1TV03HAKC	Immobilization, radius and ulna using percutaneous external fixator
Fractures	1TV03HATA	Immobilization, radius and ulna using skeletal traction
Fractures	1TV73JA	Reduction, radius and ulna using closed (external) approach
Fractures	1TV73LA	Reduction, radius and ulna using open approach
Fractures	1TV74HAKD	Fixation, radius and ulna percutaneous approach [e.g. with closed or no reduction] no tissue used using wire, mesh, staple
Fractures	1TV74HALQ	Fixation, radius and ulna percutaneous approach [e.g. with closed or no reduction] no tissue used using intramedullary nail
Fractures	1TV74HANV	Fixation, radius and ulna percutaneous approach [e.g. with closed or no reduction] no tissue used using pin, nail

FRACTURES	1TV74HANVN	Fixation, radius and ulna percutaneous approach [e.g. with closed or no reduction] with synthetic tissue [e.g. bone cement or paste] using pin, nail
Fractures	1TV74HANW	Fixation, radius and ulna percutaneous approach [e.g. with closed or no reduction] no tissue used using plate, screw
Fractures	1TV74LA	Fixation, radius and ulna open approach no tissue used using no fixative device (e.g. for epiphysiodesis)
Fractures	1TV74LAKD	Fixation, radius and ulna open approach no tissue used using wire, mesh, staple
Fractures	1TV74LAKDA	Fixation, radius and ulna open approach with bone autograft using wire, mesh, staple
Fractures	1TV74LAKDK	Fixation, radius and ulna open approach with bone homograft using wire, mesh, staple
Fractures	1TV74LAKDN	Fixation, radius and ulna open approach with synthetic tissue [e.g. bone cement or paste] using wire, mesh, staple
Fractures	1TV74LAKDQ	Fixation, radius and ulna open approach with combined sources of tissue [e.g. bone graft, cement/paste] using wire, mesh, staple
Fractures	1TV74LALQ	Fixation, radius and ulna open approach no tissue used using intramedullary nail
Fractures	1TV74LALQA	Fixation, radius and ulna open approach with bone autograft using intramedullary nail
Fractures	1TV74LALQK	Fixation, radius and ulna open approach with bone homograft using intramedullary nail
Fractures	1TV74LALQN	Fixation, radius and ulna open approach with synthetic tissue [e.g. bone cement or paste] using intramedullary nail
Fractures	1TV74LALQQ	Fixation, radius and ulna open approach with combined sources of tissue [e.g. bone graft, cement/paste] using intramedullary nail
Fractures	1TV74LANV	Fixation, radius and ulna open approach no tissue used using pin, nail
Fractures	1TV74LANVA	Fixation, radius and ulna open approach with bone autograft using pin, nail
Fractures	1TV74LANVK	Fixation, radius and ulna open approach with bone homograft using pin, nail
Fractures	1TV74LANVN	Fixation, radius and ulna open approach with synthetic tissue [e.g. bone cement or paste] using pin, nail
Fractures	1TV74LANVQ	Fixation, radius and ulna open approach with combined sources of tissue [e.g. bone graft, cement/paste] using pin, nail
Fractures	1TV74LANW	Fixation, radius and ulna open approach no tissue used using plate, screw
Fractures	1TV74LANWA	Fixation, radius and ulna open approach with bone autograft using plate, screw
Fractures	1TV74LANWK	Fixation, radius and ulna open approach with bone homograft using plate, screw
Fractures	1TV74LANWN	Fixation, radius and ulna open approach with synthetic tissue [e.g. bone cement or paste] using plate, screw
Fractures	1TV74LANWQ	Fixation, radius and ulna open approach with combined sources of tissue [e.g. bone graft, cement/paste] using plate, screw

Fractures	1UB74HAKDN	Fixation, wrist joint using percutaneous approach [e.g. with closed reduction or no reduction] using wire, mesh, staple with synthetic tissue [e.g. bone cement or paste]
Fractures	1UB74HANW	Fixation, wrist joint percutaneous approach [e.g. with closed or no reduction] using plate, screw
Fractures	1UE73JA	Reduction, first metacarpal bone using closed (external) approach
Fractures	1UE73LA	Reduction, first metacarpal bone using open approach
Fractures	1UE74HAKD	Fixation, first metacarpal bones percutaneous approach [e.g. with closed or no reduction] fixation device alone using wire, staple
Fractures	1UE74HANV	Fixation, first metacarpal bones percutaneous approach [e.g. with closed or no reduction] fixation device alone using pin, nail
Fractures	1UE74LAKD	Fixation, first metacarpal bones open approach fixation device alone using wire, staple
Fractures	1UE74LAKDA	Fixation, first metacarpal bones open approach with bone autograft using wire, staple
Fractures	1UE74LAKDK	Fixation, first metacarpal bones open approach with bone homograft using wire, staple
Fractures	1UE74LAKDN	Fixation, first metacarpal bones open approach with synthetic tissue [e.g. bone cement or paste] using wire, staple
Fractures	1UE74LAKDQ	Fixation, first metacarpal bones open approach with combined sources of tissue [e.g. bone graft, cement/paste] using wire, staple
Fractures	1UE74LALQ	Fixation, first metacarpal bones open approach fixation device alone using intramedullary nail
Fractures	1UE74LALQA	Fixation, first metacarpal bones open approach with bone autograft using intramedullary nail
Fractures	1UE74LALQK	Fixation, first metacarpal bones open approach with bone homograft using intramedullary nail
Fractures	1UE74LALQN	Fixation, first metacarpal bones open approach with synthetic tissue [e.g. bone cement or paste] using intramedullary nail
Fractures	1UE74LALQQ	Fixation, first metacarpal bones open approach with combined sources of tissue [e.g. bone graft, cement/paste] using intramedullary nail
Fractures	1UE74LANV	Fixation, first metacarpal bones open approach fixation device alone using pin, nail
Fractures	1UE74LANVA	Fixation, first metacarpal bones open approach with bone autograft using pin, nail
Fractures	1UE74LANVK	Fixation, first metacarpal bones open approach with bone homograft using pin, nail
Fractures	1UE74LANVN	Fixation, first metacarpal bones open approach with synthetic tissue [e.g. bone cement or paste] using pin, nail
Fractures	1UE74LANVQ	Fixation, first metacarpal bones open approach with combined sources of tissue [e.g. bone graft, cement/paste] using pin, nail
Fractures	1UE74LANW	Fixation, first metacarpal bones open approach fixation device alone using plate, screw
Fractures	1UE74LANWA	Fixation, first metacarpal bones open approach with bone autograft using plate, screw
Fractures	1UE74LANWK	Fixation, first metacarpal bones open approach with bone homograft using plate, screw

Fractures	1UE74LANWN	Fixation, metacarpal bones open approach with synthetic tissue [e.g. bone cement or paste] using plate, screw
Fractures	1UE74LANWQ	Fixation, first metacarpal bones open approach with combined sources of tissue [e.g. bone graft, cement/paste] using plate, screw
Fractures	1UF73JA	Reduction, other metacarpal bones using closed (external) approach
Fractures	1UF73LA	Reduction, other metacarpal bones using open approach
Fractures	1UF74HAKD	Fixation, other metacarpal bones percutaneous approach [e.g. with closed or no reduction] fixation device alone using wire, staple
Fractures	1UF74HALQ	Fixation, other metacarpal bones percutaneous approach [e.g. with closed or no reduction] fixation device alone using intramedullary nail
Fractures	1UF74HANV	Fixation, other metacarpal bones percutaneous approach [e.g. with closed or no reduction] fixation device alone using pin, nail
Fractures	1UF74HANW	Fixation, other metacarpal bones percutaneous approach [e.g. with closed or no reduction] fixation device alone using plate, screw
Fractures	1UF74LAKD	Fixation, other metacarpal bones open approach fixation device alone using wire, staple
Fractures	1UF74LAKDA	Fixation, other metacarpal bones open approach with bone autograft using wire, staple
Fractures	1UF74LAKDK	Fixation, other metacarpal bones open approach with bone homograft using wire, staple
Fractures	1UF74LAKDN	Fixation, other metacarpal bones open approach with synthetic tissue [e.g. bone cement or paste] using wire, staple
Fractures	1UF74LAKDQ	Fixation, other metacarpal bones open approach with combined bone graft and cement, paste using wire, staple
Fractures	1UF74LALQ	Fixation, other metacarpal bones open approach fixation device alone using intramedullary nail
Fractures	1UF74LALQA	Fixation, other metacarpal bones open approach with bone autograft using intramedullary nail
Fractures	1UF74LALQK	Fixation, other metacarpal bones open approach with bone homograft using intramedullary nail
Fractures	1UF74LALQN	Fixation, other metacarpal bones open approach with synthetic tissue [e.g. bone cement or paste] using intramedullary nail
Fractures	1UF74LALQQ	Fixation, other metacarpal bones open approach with combined sources of tissue [e.g. graft & cement/paste] using intramedullary nail
Fractures	1UF74LANV	Fixation, other metacarpal bones open approach fixation device alone using pin, nail
Fractures	1UF74LANVA	Fixation, other metacarpal bones open approach with bone autograft using pin, nail
Fractures	1UF74LANVK	Fixation, other metacarpal bones open approach with bone homograft using pin, nail
Fractures	1UF74LANVN	Fixation, other metacarpal bones open approach with synthetic tissue [e.g. bone cement or paste] using pin, nail
Fractures	1UF74LANVQ	Fixation, other metacarpal bones open approach with combined sources of tissue [e.g. graft & cement/paste] using pin, nail

Fractures	1UF74LANW	Fixation, other metacarpal bones open approach fixation device alone using plate, screw
Fractures	1UF74LANWA	Fixation, other metacarpal bones open approach with bone autograft using plate, screw
Fractures	1UF74LANWK	Fixation, other metacarpal bones open approach with bone homograft using plate, screw
Fractures	1UF74LANWN	Fixation, other metacarpal bones open approach with synthetic tissue [e.g. bone cement or paste] using plate, screw
Fractures	1UF74LANWQ	Fixation, other metacarpal bones open approach with combined sources of tissue [e.g. graft & cement/paste] using plate, screw
Fractures	1UG74HANW	Fixation, other metacarpophalangeal joint(s) percutaneous approach [e.g. with closed or no reduction] fixation device alone using plate, screw
Fractures	1UG74LAKDA	Fixation, other metacarpophalangeal joint(s) open approach, with bone autograft using wire, staple, tension band
Fractures	1UG74LAKDN	Fixation, other metacarpophalangeal joint(s) open approach, with synthetic material [bone cement, paste] using wire, staple, tension band
Fractures	1UG74LAKDQ	Fixation, other metacarpophalangeal joint(s) open approach, with combined sources of tissue [e.g. bone graft, cement/paste] using wire, staple, tension band
Fractures	1UG74LALQA	Fixation, other metacarpophalangeal joint(s) open approach, with bone autograft using intramedullary nail
Fractures	1UG74LALQN	Fixation, other metacarpophalangeal joint(s) open approach with synthetic material [bone cement, paste] using intramedullary nail
Fractures	1UG74LALQQ	Fixation, other metacarpophalangeal joint(s) open approach with combined sources of tissue [e.g. bone graft, cement/paste] using intramedullary nail
Fractures	1UG74LANVA	Fixation, other metacarpophalangeal joint(s) open approach with bone autograft using pin, nail
Fractures	1UG74LANVN	Fixation, other metacarpophalangeal joint(s) open approach with synthetic material [bone cement, paste] using pin, nail
Fractures	1UG74LANVQ	Fixation, other metacarpophalangeal joint(s) open approach with combined sources of tissue [e.g. bone graft, cement/paste] using pin, nail
Fractures	1UG74LANWA	Fixation, other metacarpophalangeal joint(s) open approach with bone autograft using plate, screw
Fractures	1UG74LANWN	Fixation, other metacarpophalangeal joint(s) open approach with synthetic material [bone cement, paste] using plate, screw
Fractures	1UG74LANWQ	Fixation, other metacarpophalangeal joint(s) open approach with combined sources of tissue [e.g. bone graft, cement/paste] using plate, screw
Fractures	1UH73JA	Reduction, first metacarpophalangeal joint using closed (external) approach
Fractures	1UH73LA	Reduction, first metacarpophalangeal joint using open approach
Fractures	1UH74HAKD	Fixation, first metacarpophalangeal joint percutaneous approach [closed], fixation device alone using wire, staple, tension band

Fractures	1UH74HALQ	Fixation, first metacarpophalangeal joint percutaneous approach [closed], fixation device alone using intramedullary nail
Fractures	1UH74HANV	Fixation, first metacarpophalangeal joint percutaneous approach [closed], fixation device alone using pin, nail
Fractures	1UH74HANW	Fixation, first metacarpophalangeal joint percutaneous approach [e.g. with closed or no reduction], fixation device alone using plate, screw
Fractures	1UH74LAKD	Fixation, first metacarpophalangeal joint open approach, fixation device alone using wire, staple, tension band
Fractures	1UH74LAKDA	Fixation, first metacarpophalangeal joint open approach with bone autograft using wire, staple, tension band
Fractures	1UH74LAKDN	Fixation, first metacarpophalangeal joint open approach, synthetic material [bone, cement, paste] using wire, staple, tension band
Fractures	1UH74LAKDQ	Fixation, first metacarpophalangeal joint open approach with combined sources of tissue [e.g. bone graft, cement/paste] using wire, staple, tension band
Fractures	1UH74LALQ	Fixation, first metacarpophalangeal joint, open approach, fixation device alone using intramedullary nail
Fractures	1UH74LALQA	Fixation, first metacarpophalangeal joint open approach, with bone autograft using intramedullary nail
Fractures	1UH74LALQN	Fixation, first metacarpophalangeal joint, open approach with synthetic material [bone cement, paste] using intramedullary nail
Fractures	1UH74LALQQ	Fixation, first metacarpophalangeal joint open approach, with combined sources of tissue [e.g. bone graft, cemet/paste] using intramedullary nail
Fractures	1UH74LANV	Fixation, first metacarpophalangeal joint open approach, fixation device alone using pin, nail
Fractures	1UH74LANVA	Fixation, first metacarpophalangeal joint using open approach with bone autograft using pin, nail
Fractures	1UH74LANVN	Fixation, first metacarpophalangeal joint open approach with synthetic material [bone, cement, paste] using pin, nail
Fractures	1UH74LANVQ	Fixation, first metacarpophalangeal joint open approach with combined souces of tissue [e.g. bone graft, cement/paste] using pin, nail
Fractures	1UH74LANW	Fixation, first metacarpophalangeal joint open approach, fixation device alone using plate, screw
Fractures	1UH74LANWA	Fixation, first metacarpophalangeal joint open approach with bone autograft using plate, screw
Fractures	1UH74LANWN	Fixation, first metacarpophalangeal joint open approach with synthetic tissue [bone, cement, paste] using plate, screw
Fractures	1UH74LANWQ	Fixation, first metacarpophalangeal joint open approach with combined sources of tissue [e.g. bone graft, cement/paste] using plate, screw
Fractures	1UI03HAKC	Immobilization, first phalanx of hand using percutaneous external fixator
Fractures	1UI73JA	Reduction, first phalanx of hand using closed (external) approach
Fractures	1UI73LA	Reduction, first phalanx of hand using open approach

Fractures	1UI74HAKD	Fixation, first phalanx of hand percutaneous approach [e.g. with closed or no reduction] fixation device alone using wire, staple, tension band
Fractures	1UI74HALQ	Fixation, first phalanx of hand percutaneous approach [e.g. with closed or no reduction] fixation device alone using intramedullary nail
Fractures	1UI74HANV	Fixation, first phalanx of hand percutaneous approach [e.g. with closed or no reduction] fixation device alone using pin, nail
Fractures	1UI74HANW	Fixation, first phalanx of hand percutaneous approach [e.g. with closed or no reduction] fixation device alone using plate, screw
Fractures	1UI74LAKD	Fixation, first phalanx of hand open approach fixation device alone using wire, staple, tension band
Fractures	1UI74LAKDA	Fixation, first phalanx of hand open approach with bone autograft using wire, staple, tension band
Fractures	1UI74LAKDN	Fixation, first phalanx of hand open approach with synthetic tissue [bone, cement, paste] using wire, staple, tension band
Fractures	1UI74LAKDQ	Fixation, first phalanx of hand open approach with combined sources of tissue [e.g. bone graft, cement/paste] using wire, staple, tension band
Fractures	1UI74LALQ	Fixation, first phalanx of hand open approach fixation device alone using intramedullary nail
Fractures	1UI74LALQA	Fixation, first phalanx of hand open approach with bone autograft using intramedullary nail
Fractures	1UI74LALQN	Fixation, first phalanx of hand open approach with synthetic tissue [bone cement, paste] using intramedullary nail
Fractures	1UI74LALQQ	Fixation, first phalanx of hand open approach with combined sources of tissue [e.g. bone graft, cement/paste] using intramedullary nail
Fractures	1UI74LANV	Fixation, first phalanx of hand open approach fixation device alone using pin, nail
Fractures	1UI74LANVA	Fixation, first phalanx of hand open approach with bone autograft using pin, nail
Fractures	1UI74LANVN	Fixation, first phalanx of hand open approach with synthetic tissue [bone, cement, paste] using pin, nail
Fractures	1UI74LANVQ	Fixation, first phalanx of hand open approach with combined sources of tissue [e.g. bone graft, cement/paste] using pin, nail
Fractures	1UI74LANW	Fixation, first phalanx of hand open approach fixation device alone using plate, screw
Fractures	1UI74LANWA	Fixation, first phalanx of hand open approach with bone autograft using plate, screw
Fractures	1UI74LANWN	Fixation, first phalanx of hand open approach with synthetic tissue [bone, cement, paste] using plate, screw
Fractures	1UI74LANWQ	Fixation, first phalanx of hand open approach with combined sources of tissue [e.g. bone graft, cement/paste] using plate, screw
Fractures	1UJ03HAKC	Immobilization, other phalanx of hand using percutaneous external fixator
Fractures	1UJ73JA	Reduction, other phalanx of hand using closed (external) approach
Fractures	1UJ73LA	Reduction, other phalanx of hand using open approach

Fractures	1UJ74HAKD	Fixation, other phalanx of hand percutaneous approach [e.g. with closed or no reduction] fixation device alone using wire, staple, tension band
Fractures	1UJ74HALQ	Fixation, other phalanx of hand percutaneous approach [e.g. with closed or no reduction] fixation device alone using intramedullary nail
Fractures	1UJ74HANV	Fixation, other phalanx of hand percutaneous approach [e.g. with closed or no reduction] fixation device alone using pin, nail
Fractures	1UJ74HANW	Fixation, other phalanx of hand percutaneous approach [e.g. with closed or no reduction] fixation device alone using plate, screw
Fractures	1UJ74LAKD	Fixation, other phalanx of hand open approach fixation device alone using wire, staple, tension band
Fractures	1UJ74LAKDA	Fixation, other phalanx of hand open approach with bone autograft using wire, staple, tension band
Fractures	1UJ74LAKDN	Fixation, other phalanx of hand open approach with synthetic tissue [bone cement, paste] using wire, staple, tension band
Fractures	1UJ74LAKDQ	Fixation, other phalanx of hand open approach with combined sources of tissue [e.g. bone graft, cement/paste] using wire, staple, tension band
Fractures	1UJ74LALQ	Fixation, other phalanx of hand open approach fixation device alone using intramedullary nail
Fractures	1UJ74LALQA	Fixation, other phalanx of hand open approach with bone autograft using intramedullary nail
Fractures	1UJ74LALQN	Fixation, other phalanx of hand open approach with synthetic material [bone cement, paste] using intramedullary nail
Fractures	1UJ74LALQQ	Fixation, other phalanx of hand open approach with combined sources of tissue [e.g. bone graft, cement/paste] using intramedullary nail
Fractures	1UJ74LANV	Fixation, other phalanx of hand open approach fixation device alone using pin, nail
Fractures	1UJ74LANVA	Fixation, other phalanx of hand open approach with bone autograft using pin, nail
Fractures	1UJ74LANVN	Fixation, other phalanx of hand open approach with synthetic tissue [bone cement, paste] using pin, nail
Fractures	1UJ74LANVQ	Fixation, other phalanx of hand open approach with combined sources of tissue [e.g. bone graft, cement/paste] using pin, nail
Fractures	1UJ74LANW	Fixation, other phalanx of hand open approach fixation device alone using plate, screw
Fractures	1UJ74LANWA	Fixation, other phalanx of hand open approach with bone autograft using plate, screw
Fractures	1UJ74LANWN	Fixation, other phalanx of hand open approach with synthetic tissue [bone cement, paste] using plate, screw
Fractures	1UJ74LANWQ	Fixation, other phalanx of hand open approach with combined sources of tissue [e.g. bone graft, cement/paste] using plate, screw
Fractures	1UK73JA	Reduction, other interphalangeal joints of hand using closed (external) approach
Fractures	1UM74HAKD	Fixation, first interphalangeal joint of hand using percutaneous (closed) approach and wire

Fractures	1UM74HANW	Fixation, first interphalangeal joint of hand using percutaneous (closed) approach and plate/screw
Fractures	1UM74LAKD	Fixation, first interphalangeal joint of hand using open approach and wire
Fractures	1UM74LANV	Fixation, first interphalangeal joint of hand using open approach and pin or nail
Fractures	1UM74LANW	Fixation, first interphalangeal joint of hand using open approach and screw
FRACTURES	1UM75LAKDA	Fusion, first interphalangeal joint of hand open approach with bone autograft using wire, tension band
FRACTURES	1UM75LANWA	Fusion, first interphalangeal joint of hand open approach with bone autograft using plate, screw device
Fractures	1VC03HAFQ	Immobilization, femur with percutaneous traction[e.g. skeletal] using cast [e.g. spica, hanged brace cast]
Fractures	1VC03HAKC	Immobilization, femur with percutaneous traction[e.g. skeletal] using percutaneous external fixator
Fractures	1VC03HASR	Immobilization, femur with percutaneous traction[e.g. skeletal] using splinting device
Fractures	1VC03HATA	Immobilization, femur with percutaneous traction[e.g. skeletal] using traction alone
Fractures	1VC73JA	Reduction, femur using closed (external) approach
Fractures	1VC73LA	Reduction, femur using open approach
Fractures	1VC74HA	Fixation, femur percutaneous approach [e.g. with closed reduction or no reduction] no tissue used using no fixative device [e.g. for epiphysiodesis]
Fractures	1VC74HALQ	Fixation, femur percutaneous approach [e.g. with closed reduction or no reduction] fixation device alone using intramedullary nail
Fractures	1VC74HANV	Fixation, femur percutaneous approach [e.g. with closed reduction or no reduction] fixation device alone using pin, nail
Fractures	1VC74HANW	Fixation, femur using percutaneous approach [e.g with closed reduction or no reduction] using plate/screw
Fractures	1VC74LA	Fixation, femur open approach using no fixative device (e.g. for epiphysiodesis)
Fractures	1VC74LAKD	Fixation, femur open approach fixation device alone using wire, staple, cable
Fractures	1VC74LAKDA	Fixation, femur open approach with bone autograft using wire, staple, cable
Fractures	1VC74LAKDK	Fixation, femur open approach with bone homograft using wire, staple, cable
Fractures	1VC74LAKDN	Fixation, femur open approach with synthetic tissue [e.g. bone cement, or paste] using wire, staple, cable
Fractures	1VC74LAKDQ	Fixation, femur open approach with combined sources of tissue [e.g. graft & cement/paste] using wire, staple, cable
Fractures	1VC74LALQ	Fixation, femur open approach fixation device alone using intramedullary nail
Fractures	1VC74LALQA	Fixation, femur open approach with bone autograft using intramedullary nail
Fractures	1VC74LALQK	Fixation, femur open approach with bone homograft using intramedullary nail
Fractures	1VC74LALQN	Fixation, femur open approach with synthetic tissue [e.g. bone cement, or paste] using intramedullary nail

Fractures	1VC74LALQQ	Fixation, femur open approach with combined sources of tissue [e.g. graft & cement/paste] using intramedullary nail
Fractures	1VC74LANV	Fixation, femur open approach fixation device alone using pin, nail
Fractures	1VC74LANVA	Fixation, femur open approach with bone autograft using pin, nail
Fractures	1VC74LANVK	Fixation, femur open approach with bone homograft using pin, nail
Fractures	1VC74LANVN	Fixation, femur open approach with synthetic tissue [e.g. bone cement, or paste] using pin, nail
Fractures	1VC74LANVQ	Fixation, femur open approach with combined sources of tissue [e.g. graft & cement/paste] using pin, nail
Fractures	1VC74LANW	Fixation, femur open approach fixation device alone using screw, plate and screw
Fractures	1VC74LANWA	Fixation, femur open approach with bone autograft using screw, plate and screw
Fractures	1VC74LANWK	Fixation, femur open approach with bone homograft using screw, plate and screw
Fractures	1VC74LANWN	Fixation, femur open approach with synthetic tissue [e.g. bone cement, or paste] using screw, plate and screw
Fractures	1VC74LANWQ	Fixation, femur open approach with combined sources of tissue [e.g. graft & cement/paste] using screw, plate and screw
Fractures	1VG74DANW	Fixation, knee joint using endoscopic [arthroscopic] approach using screw, plate and screw
Fractures	1VP73JA	Reduction, patella using closed [external] approach
Fractures	1VP73LA	Reduction, patella using open approach
Fractures	1VP74HANW	Fixation, patella using percutaneous approach and plate, screw
Fractures	1VP74LAKD	Fixation, patella using open approach and wire, tension band (encirclage)
Fractures	1VP74LAKDN	Fixation, patella using open approach and synthetic tissue with wire, tension band
Fractures	1VP74LANW	Fixation, patella using open approach and screw/plate
Fractures	1VP74LAXXN	Fixation, patella using open approach and synthetic tissue [e.g. bone cement or paste]
Fractures	1VQ03HAFQ	Immobilization, tibia and fibula with percutaneous traction[e.g. skeletal] using cast [e.g. support, weight bearing]
Fractures	1VQ03HAKC	Immobilization, tibia and fibula with percutaneous traction[e.g. skeletal] using external fixator [percutaneous pin, wire]
Fractures	1VQ03HASR	Immobilization, tibia and fibula with percutaneous traction[e.g. skeletal] using splinting device
Fractures	1VQ03HATA	Immobilization, tibia and fibula with percutaneous traction[e.g. skeletal] using traction alone
Fractures	1VQ73JA	Reduction, tibia and fibula using closed (external) approach
Fractures	1VQ73LA	Reduction, tibia and fibula using open approach
Fractures	1VQ74HA	Fixation, tibia and fibula percutaneous approach [e.g. with closed or no reduction] no tissue used using no fixative device [e.g. for epiphysiodesis]

Fractures	1VQ74HALQ	Fixation, tibia and fibula percutaneous approach [e.g. with closed or no reduction] no tissue used using intramedullary nail
Fractures	1VQ74HANV	Fixation, tibia and fibula percutaneous approach [e.g. with closed or no reduction] no tissue used using pin, nail
Fractures	1VQ74LA	Fixation, tibia and fibula open approach no tissue used using no fixative device (e.g. for epiphysiodesis)
Fractures	1VQ74LAKD	Fixation, tibia and fibula open approach no tissue used using wire, mesh, staple
Fractures	1VQ74LAKDA	Fixation, tibia and fibula open approach with bone autograft using wire, mesh, staple
Fractures	1VQ74LAKDK	Fixation, tibia and fibula open approach with bone homograft using wire, mesh, staple
Fractures	1VQ74LAKDN	Fixation, tibia and fibula open approach with synthetic tissue [e.g. bone cement, or paste] using wire, mesh, staple
Fractures	1VQ74LAKDQ	Fixation, tibia and fibula open approach with combined bone graft and cement, or paste using wire, mesh, staple
Fractures	1VQ74LALQ	Fixation, tibia and fibula open approach no tissue used using intramedullary nail
Fractures	1VQ74LALQA	Fixation, tibia and fibula open approach with bone autograft using intramedullary nail
Fractures	1VQ74LALQK	Fixation, tibia and fibula open approach with bone homograft using intramedullary nail
Fractures	1VQ74LALQN	Fixation, tibia and fibula open approach with synthetic tissue [e.g. bone cement, or paste] using intramedullary nail
Fractures	1VQ74LALQQ	Fixation, tibia and fibula open approach with combined bone graft and cement, or paste using intramedullary nail
Fractures	1VQ74LANV	Fixation, tibia and fibula open approach no tissue used using pin, nail
Fractures	1VQ74LANVA	Fixation, tibia and fibula open approach with bone autograft using pin, nail
Fractures	1VQ74LANVK	Fixation, tibia and fibula open approach with bone homograft using pin, nail
Fractures	1VQ74LANVN	Fixation, tibia and fibula open approach with synthetic tissue [e.g. bone cement, or paste] using pin, nail
Fractures	1VQ74LANVQ	Fixation, tibia and fibula open approach with combined bone graft and cement, or paste using pin, nail
Fractures	1VQ74LANW	Fixation, tibia and fibula open approach no tissue used using plate, screw
Fractures	1VQ74LANWA	Fixation, tibia and fibula open approach with bone autograft using plate, screw
Fractures	1VQ74LANWK	Fixation, tibia and fibula open approach with bone homograft using plate, screw
Fractures	1VQ74LANWN	Fixation, tibia and fibula open approach with synthetic tissue [e.g. bone cement, or paste] using plate, screw
Fractures	1VQ74LANWQ	Fixation, tibia and fibula open approach with combined bone graft and cement, or paste using plate, screw
Fractures	1WE73JA	Reduction, tarsal bones and intertarsal joints [hindfoot, midfoot] using closed (external) approach
Fractures	1WE73LA	Reduction, tarsal bones and intertarsal joints [hindfoot, midfoot] using open approach

Fractures	1WE74HAKD	Fixation, tarsal bones and intertarsal joints [hindfoot, midfoot] percutaneous approach using wire, staple, tension band
Fractures	1WE74HANV	Fixation, tarsal bones and intertarsal joints [hindfoot, midfoot] percutaneous approach using pin, nail
Fractures	1WE74HANW	Fixation, tarsal bones and intertarsal joints [hindfoot, midfoot] percutaneous approach using screw, plate and screw
Fractures	1WE74LAKD	Fixation, tarsal bones and intertarsal joints [hindfoot, midfoot] open approach using wire, staple, tension band
Fractures	1WE74LAKDA	Fixation, tarsal bones and intertarsal joints [hindfoot, midfoot] open approach using wire, staple, tension band and autograft
Fractures	1WE74LAKDK	Fixation, tarsal bones and intertarsal joints [hindfoot, midfoot] open approach with homograft using wire, staple, tension band
Fractures	1WE74LAKDN	Fixation, tarsal bones and intertarsal joints [hindfoot, midfoot] open approach with synthetic tissue [e.g. bone cement, or paste] using wire, staple, tension band
Fractures	1WE74LAKDQ	Fixation, tarsal bones and intertarsal joints [hindfoot, midfoot] open approach with combined sources of tissue [e.g. bone graft, cement/paste] using wire, staple, tension band
Fractures	1WE74LANV	Fixation, tarsal bones and intertarsal joints [hindfoot, midfoot] open approach using pin, nail
Fractures	1WE74LANVA	Fixation, tarsal bones and intertarsal joints [hindfoot, midfoot] open approach using pin, nail and autograft
Fractures	1WE74LANVK	Fixation, tarsal bones and intertarsal joints [hindfoot, midfoot] open approach with homograft using pin, nail
Fractures	1WE74LANVN	Fixation, tarsal bones and intertarsal joints [hindfoot, midfoot] open approach with synthetic tissue [e.g. bone cement, or paste] using pin, nail
Fractures	1WE74LANVQ	Fixation, tarsal bones and intertarsal joints [hindfoot, midfoot] open approach with combined sources of tissue [e.g. bone graft, cement/paste] using pin, nail
Fractures	1WE74LANW	Fixation, tarsal bones and intertarsal joints [hindfoot, midfoot] open approach using screw, plate and screw
Fractures	1WE74LANWA	Fixation, tarsal bones and intertarsal joints [hindfoot, midfoot] open approach using screw, plate and screw and autograft
Fractures	1WE74LANWK	Fixation, tarsal bones and intertarsal joints [hindfoot, midfoot] open approach with homograft using screw, plate and screw
Fractures	1WE74LANWN	Fixation, tarsal bones and intertarsal joints [hindfoot, midfoot] open approach with synthetic tissue [e.g. bone cement, or paste] using screw, plate and screw
Fractures	1WE74LANWQ	Fixation, tarsal bones and intertarsal joints [hindfoot, midfoot] open approach with combined sources of tissue [e.g. bone graft, cement/paste] using screw, plate and screw
Fractures	1WI73JA	Reduction, first metatarsal bone and first metatarsophalangeal joint using closed (external) approach
Fractures	1WI73LA	Reduction, first metatarsal bone and first metatarsophalangeal joint using open approach

Fractures	1WI74HAKD	Fixation, first metatarsal bone and first metatarsophalangeal joint using percutaneous approach using wire, staple, tension band
Fractures	1WI74HANV	Fixation, first metatarsal bone and first metatarsophalangeal joint percutaneous approach using pin, nail
Fractures	1WI74HANW	Fixation, first metatarsal bone and first metatarsophalangeal joint percutaneous approach using plate and screw
Fractures	1WI74LAKD	Fixation, first metatarsal bone and first metatarsophalangeal joint open approach using wire, staple, tension band
Fractures	1WI74LAKDA	Fixation, first metatarsal bone and first metatarsophalangeal joint open approach and autograft using wire, staple, tension band
Fractures	1WI74LANV	Fixation, first metatarsal bone and first metatarsophalangeal joint open approach using pin, nail
Fractures	1WI74LANVA	Fixation, first metatarsal bone and first metatarsophalangeal joint open approach and auograft using pin, nail
Fractures	1WI74LANW	Fixation, first metatarsal bone and first metatarsophalangeal joint open approach using plate and screw
Fractures	1WI74LANWA	Fixation, first metatarsal bone and first metatarsophalangeal joint open approach and autograft using plate and screw
Fractures	1WJ73JA	Reduction, tarsometatarsal joints, other metatarsal bones and other metatarsophalangeal joints [forefoot] using closed (external) approach
Fractures	1WJ73LA	Reduction, tarsometatarsal joints, other metatarsal bones and other metatarsophalangeal joints [forefoot] using open approach
Fractures	1WJ74HAKD	Fixation, tarsometatarsal joints, other metatarsal bones and other metatarsophalangeal joints [forefoot] percutaneous approach using wire, staple, tension band
Fractures	1WJ74HANV	Fixation, tarsometatarsal joints, other metatarsal bones and other metatarsophalangeal joints [forefoot] percutaneous approach using pin, nail
Fractures	1WJ74HANW	Fixation, tarsometatarsal joints, other metatarsal bones and other metatarsophalangeal joints [forefoot] percutaneous approach using screw, plate and screw
Fractures	1WJ74LAKD	Fixation, tarsometatarsal joints, other metatarsal bones and other metatarsophalangeal joints [forefoot] open approach using wire, staple, tension band
Fractures	1WJ74LAKDA	Fixation, tarsometatarsal joints, other metatarsal bones and other metatarsophalangeal joints [forefoot] open approach and autograft using wire, staple, tension band
Fractures	1WJ74LAKDN	Fixation, tarsometatarsal joints, other metatarsal bones and other metatarsophalangeal joints [forefoot] open approach with synthetic tissue using wire, staple, tension band
Fractures	1WJ74LANV	Fixation, tarsometatarsal joints, other metatarsal bones and other metatarsophalangeal joints [forefoot] open approach using pin, nail

Fractures	1WJ74LANVA	Fixation, tarsometatarsal joints, other metatarsal bones and other metatarsophalangeal joints [forefoot] open approach and autograft using pin, nail
Fractures	1WJ74LANVN	Fixation, tarsometatarsal joints, other metatarsal bones and other metatarsophalangeal joints [forefoot] open approach with synthetic tissue using pin, nail
Fractures	1WJ74LANW	Fixation, tarsometatarsal joints, other metatarsal bones and other metatarsophalangeal joints [forefoot] open approach using screw, plate and screw
Fractures	1WJ74LANWA	Fixation, tarsometatarsal joints, other metatarsal bones and other metatarsophalangeal joints [forefoot] open approach and autograft using screw, plate and screw
Fractures	1WJ74LANWN	Fixation, tarsometatarsal joints, other metatarsal bones and other metatarsophalangeal joints [forefoot] open approach with synthetic tissue using screw, plate and screw
Fractures	1WK03HAKC	Immobilization, first phalanx of foot using percutaneous external fixator
Fractures	1WK74HAKD	Fixation, first phalanx of foot, percutaneous approach [e.g. with closed or no reduction] using wire, staple
Fractures	1WK74HANV	Fixation, first phalanx of foot, percutaneous approach [e.g. with closed or no reduction] using pin, nail
Fractures	1WK74HANW	Fixation, first phalanx of foot, percutaneous approach [e.g. with closed or no reduction] using screw, plate and screw
Fractures	1WK74LAKD	Fixation, first phalanx of foot, open approach using wire, staple
Fractures	1WK74LANV	Fixation, first phalanx of foot, open approach using pin, nail
Fractures	1WK74LANW	Fixation, first phalanx of foot, open approach using screw, plate and screw
Fractures	1WL03HAKC	Immobilization, other phalanx of foot using percutaneous external fixator
Fractures	1WL74HAKD	Fixation, other phalanx of foot percutaneous approach [e.g. with closed or no reduction] using wire, staple
Fractures	1WL74HANV	Fixation, other phalanx of foot percutaneous approach [e.g. with closed or no reduction] using pin, nail
Fractures	1WL74HANW	Fixation, other phalanx of foot percutaneous approach [e.g. with closed or no reduction] using screw, plate and screw
Fractures	1WL74LAKD	Fixation, other phalanx of foot open approach using wire, staple
Fractures	1WL74LANV	Fixation, other phalanx of foot open approach using pin, nail
Fractures	1WL74LANW	Fixation, other phalanx of foot open approach using screw, plate and screw
Hernia	1SY80DA	Repair, muscles of the chest and abdomen endoscopic [laparoscopic] approach without tissue [e.g. suturing or stapling]
Hernia	1SY80DAXXA	Repair, muscles of the chest and abdomen endoscopic [laparoscopic] approach using autograft [e.g. fascia, skin]
Hernia	1SY80DAXXF	Repair, muscles of the chest and abdomen endoscopic [laparoscopic] approach using free flap [e.g. free myocutaneous flap]

Hernia	1SY80DAXXG	Repair, muscles of the chest and abdomen endoscopic [laparoscopic] approach using pedicled flap [e.g. abdominis rectus or deltopectoral]
Hernia	1SY80DAXXL	Repair, muscles of the chest and abdomen, endoscopic [laparoscopic] approach using xenograft [e.g. Surgis, SIS (small intestine submucosa)]
Hernia	1SY80DAXXN	Repair, muscles of the chest and abdomen endoscopic [laparoscopic] approach using synthetic tissue [e.g. mesh, sponge]
Hernia	1SY80GDXXN	Repair, muscles of the chest and abdomen endoscopic [laparoscopic] approach with special incisional technique using synthetic tissue [e.g. mesh, sponge]
Hernia	1SY80LA	Repair, muscles of the chest and abdomen open approach without tissue [e.g. suturing or stapling]
Hernia	1SY80LAFF	Repair, muscles of the chest and abdomen open approach and using temporary abdominal closure device
Hernia	1SY80LATZ	Repair, muscles of the chest and abdomen open approach using zipper [temporary] (for repeat access to abdomen)
Hernia	1SY80LAXXA	Repair, muscles of the chest and abdomen open approach using autograft [e.g. fascia, skin]
Hernia	1SY80LAXXF	Repair, muscles of the chest and abdomen open approach using free flap [e.g. free myocutaneous flap]
Hernia	1SY80LAXXG	Repair, muscles of the chest and abdomen open approach using pedicled flap [e.g. abdominis rectus or deltopectoral]
Hernia	1SY80LAXXK	Repair, muscles of the chest and abdomen open approach using homograft [e.g. tissue from deceased donor]
Hernia	1SY80LAXXL	Repair, muscles of the chest and abdomen, open approach using xenograft [e.g. Surgisis, SIS (small intestine submucosa)]
Hernia	1SY80LAXXN	Repair, muscles of the chest and abdomen open approach using synthetic tissue [e.g. mesh, sponge]
Hernia	1SY80LAXXQ	Repair, muscles of the chest and abdomen open approach and combined sources of tissue (e.g. mesh with autograft)
Hernia	1SY80WJ	Repair, muscles of the chest and abdomen open approach using special excisional technique
Hip replacement surgery	1SQ53LAPM	Implantation of internal device, pelvis uncemented prosthetic device, single component [e.g. cup]
Hip replacement surgery	1SQ53LAPMA	Implantation of internal device, pelvis using bone autograft (uncemented) prosthetic device, single component [e.g. cup]
Hip replacement surgery	1SQ53LAPMK	Implantation of internal device, pelvis using bone homograft (uncemented) prosthetic device, single component [e.g. cup]
Hip replacement surgery	1SQ53LAPMN	Implantation of internal device, pelvis using synthetic tissue [e.g. bone cement or paste] prosthetic device, single component [e.g. cup]
Hip replacement surgery	1SQ53LAPMQ	Implantation of internal device, pelvis using combined sources of tissue [e.g. bone graft, cement/paste] prosthetic device, single component [e.g. cup]

Hip replacement surgery	1SQ53LAPN	Implantation of internal device, pelvis uncemented prosthetic device, dual component [e.g. cup with protrusio ring or additional screw, plate fixation]
Hip replacement surgery	1SQ53LAPNA	Implantation of internal device, pelvis using bone autograft (uncemented) prosthetic device, dual component [e.g. cup with protrusio ring or additional screw, plate fixation]
Hip replacement surgery	1SQ53LAPNK	Implantation of internal device, pelvis using bone homograft (uncemented) prosthetic device, dual component [e.g. cup with protrusio ring or additional screw, plate fixation]
Hip replacement surgery	1SQ53LAPNN	Implantation of internal device, pelvis using synthetic tissue [e.g. bone cement or paste] prosthetic device, dual component [e.g. cup with protrusio ring or additional screw, plate fixation]
Hip replacement surgery	1SQ53LAPNQ	Implantation of internal device, pelvis using combined sources of tissue [e.g. bone graft, cement/paste] prosthetic device, dual component [e.g. cup with protrusio ring or additional screw, plate fixation]
Hip replacement surgery	1VA53LAPM	Implantation of internal device, hip joint open approach (direct lateral, posterolateral, posterior, transgluteal) uncemented single component prosthetic device [femoral]
Hip replacement surgery	1VA53LAPMA	Implantation of internal device, hip joint open approach (direct lateral, posterolateral, posterior, transgluteal) using bone autograft [uncemented] single component prosthetic device [femoral]
Hip replacement surgery	1VA53LAPMK	Implantation of internal device, hip joint open approach (direct lateral, posterolateral, posterior, transgluteal) using bone homograft [uncemented] single component prosthetic device [femoral]
Hip replacement surgery	1VA53LAPMN	Implantation of internal device, hip joint open approach (direct lateral, posterolateral, posterior, transgluteal) using synthetic material (e.g. bone paste, cement, Dynagraft, Osteoset) single component prosthetic device [femoral]
Hip replacement surgery	1VA53LAPMQ	Implantation of internal device, hip joint open approach (direct lateral, posterolateral, posterior, transgluteal) using combined sources of tissue (e.g. bone graft, cement, paste) single component prosthetic device [femoral]
Hip replacement surgery	1VA53LAPN	Implantation of internal device, hip joint open approach (direct lateral, posterolateral, posterior, transgluteal) uncemented dual component prosthetic device [femoral with acetabular]
Hip replacement surgery	1VA53LAPNA	Implantation of internal device, hip joint open approach (direct lateral, posterolateral, posterior, transgluteal) using bone autograft [uncemented] dual component prosthetic device [femoral with acetabular]
Hip replacement surgery	1VA53LAPNK	Implantation of internal device, hip joint open approach (direct lateral, posterolateral, posterior, transgluteal) using bone homograft [uncemented] dual component prosthetic device [femoral with acetabular]
Hip replacement surgery	1VA53LAPNN	Implantation of internal device, hip joint open approach (direct lateral, posterolateral, posterior, transgluteal) using synthetic material (e.g. bone paste, cement, Dynagraft, Osteoset) dual component prosthetic device [femoral with acetabular]

Hip replacement surgery	1VA53LAPNQ	Implantation of internal device, hip joint open approach (direct lateral, posterolateral, posterior, transgluteal) using combined sources of tissue (e.g. bone graft, cement, paste) dual component prosthetic device [femoral with acetabular]
Hip replacement surgery	1VA53LASLN	Implantation of internal device, hip joint open approach (direct lateral, posterolateral, posterior, transgluteal) using synthetic material (e.g. bone paste, cement, Dynagraft, Osteoset) cement spacer [temporary, impregnated with antibiotics]
Hip replacement surgery	1VA53LLPM	Implantation of internal device, hip joint open anterior (muscle sparing) approach (anterolateral, direct anterior) uncemented single component prosthetic device [femoral]
Hip replacement surgery	1VA53LLPMA	Implantation of internal device, hip joint open anterior (muscle sparing) approach (anterolateral, direct anterior) using bone autograft [uncemented] single component prosthetic device [femoral]
Hip replacement surgery	1VA53LLPMK	Implantation of internal device, hip joint open anterior (muscle sparing) approach (anterolateral, direct anterior) using bone homograft [uncemented] single component prosthetic device [femoral]
Hip replacement surgery	1VA53LLPMN	Implantation of internal device, hip joint open anterior (muscle sparing) approach (anterolateral, direct anterior) using synthetic material (e.g. bone paste, cement, Dynagraft, Osteoset) single component prosthetic device [femoral]
Hip replacement surgery	1VA53LLPMQ	Implantation of internal device, hip joint open anterior (muscle sparing) approach (anterolateral, direct anterior) using combined sources of tissue (e.g. bone graft, cement, paste) single component prosthetic device [femoral]
Hip replacement surgery	1VA53LLPN	Implantation of internal device, hip joint open anterior (muscle sparing) approach (anterolateral, direct anterior) uncemented dual component prosthetic device [femoral with acetabular]
Hip replacement surgery	1VA53LLPNA	Implantation of internal device, hip joint open anterior (muscle sparing) approach (anterolateral, direct anterior) using bone autograft [uncemented] dual component prosthetic device [femoral with acetabular]
Hip replacement surgery	1VA53LLPNK	Implantation of internal device, hip joint open anterior (muscle sparing) approach (anterolateral, direct anterior) using bone homograft [uncemented] dual component prosthetic device [femoral with acetabular]
Hip replacement surgery	1VA53LLPNN	Implantation of internal device, hip joint open anterior (muscle sparing) approach (anterolateral, direct anterior) using synthetic material (e.g. bone paste, cement, Dynagraft, Osteoset) dual component prosthetic device [femoral with acetabular]
Hip replacement surgery	1VA53LLPNQ	Implantation of internal device, hip joint open anterior (muscle sparing) approach (anterolateral, direct anterior) using combined sources of tissue (e.g. bone graft, cement, paste) dual component prosthetic device [femoral with acetabular]
Hip replacement surgery	1VA53LLSLN	Implantation of internal device, hip joint open anterior (muscle sparing) approach (anterolateral, direct anterior)

		using synthetic material (e.g. bone paste, cement, Dynagraft, Osteoset) cement spacer [temporary, impregnated with antibiotics]
Hip replacement surgery	1VA80LAKDA	Repair, hip joint open approach with wire using autograft [e.g. bone or tendon]
Hip replacement surgery	1VA80LAKDF	Repair, hip joint open approach with wire using free flap [free fibular vascularized flap]
Hip replacement surgery	1VA80LAKDG	Repair, hip joint open approach with wire using pedicled flap [bone on vascular pedicle from ilium]
Hip replacement surgery	1VA80LAKDK	Repair, hip joint open approach with wire using homograft [e.g. bone or tendon]
Hip replacement surgery	1VA80LAXXA	Repair, hip joint open approach using autograft [e.g. bone or tendon]
Hip replacement surgery	1VA80LAXXF	Repair, hip joint open approach using free flap [free fibular vascularized flap]
Hip replacement surgery	1VA80LAXXG	Repair, hip joint open approach using pedicled flap [bone on vascular pedicle from ilium]
Hip replacement surgery	1VA80LAXXK	Repair, hip joint open approach using homograft [e.g. bone or tendon]
Hip replacement surgery	1VA80LAXXN	Repair, hip joint open approach using synthetic tissue [e.g. gortex, artificial polymer cartilage]
Hysterectomy	1RM87CAGX	Excision partial, uterus and surrounding structures per orifice [transvaginal] approach using device NEC
Hysterectomy	1RM87LAGX	Excision partial, uterus and surrounding structures open approach using device NEC
Hysterectomy	1RM89AA	Excision total, uterus and surrounding structures using combined laparoscopic and vaginal approach
Hysterectomy	1RM89CA	Excision total, uterus and surrounding structures using vaginal approach
Hysterectomy	1RM89DA	Excision total, uterus and surrounding structures using endoscopic (laparoscopic) approach
Hysterectomy	1RM89LA	Excision total, uterus and surrounding structures using open approach
Hysterectomy	1RM91AA	Excision radical, uterus and surrounding structures using combined laparoscopic and vaginal approach
Hysterectomy	1RM91CA	Excision radical, uterus and surrounding structures using vaginal approach (e.g. Schauta operation)
Hysterectomy	1RM91DA	Excision radical, uterus and surrounding structures using endoscopic (laparoscopic) approach
Hysterectomy	1RM91LA	Excision radical, uterus and surrounding structures using abdominal approach (e.g. Wertheim operation)
Hysterectomy	5CA89CK	Surgical termination of pregnancy, hysterectomy vaginal approach
Hysterectomy	5CA89GB	Surgical termination of pregnancy, endoscopic approach hysterectomy
Hysterectomy	5CA89WJ	Surgical termination of pregnancy, open approach hysterectomy
Knee replacement surgery	1VG53LAPM	Implantation of internal device, knee joint uncemented single component prosthetic device
Knee replacement surgery	1VG53LAPMA	Implantation of internal device, knee joint with bone autograft single component prosthetic device
Knee replacement surgery	1VG53LAPMK	Implantation of internal device, knee joint with bone homograft single component prosthetic device

Knee replacement surgery	1VG53LAPMN	Implantation of internal device, knee joint with synthetic material (e.g. bone paste, cement, Dynagraft, Osteoset) single component prosthetic device
Knee replacement surgery	1VG53LAPMQ	Implantation of internal device, knee joint with combined sources of tissue (e.g. bone graft, cement, paste) single component prosthetic device
Knee replacement surgery	1VG53LAPN	Implantation of internal device, knee joint uncemented using dual component prosthetic device
Knee replacement surgery	1VG53LAPNA	Implantation of internal device, knee joint with bone autograft dual component prosthetic device
Knee replacement surgery	1VG53LAPNK	Implantation of internal device, knee joint with bone homograft dual component prosthetic device
Knee replacement surgery	1VG53LAPNN	Implantation of internal device, knee joint with synthetic material (e.g. bone paste, cement, Dynagraft, Osteoset) dual component prosthetic device
Knee replacement surgery	1VG53LAPNQ	Implantation of internal device, knee joint with combined sources of tissue (e.g. bone graft, cement, paste) dual component prosthetic device
Knee replacement surgery	1VG53LAPP	Implantation of internal device, knee joint uncemented tri component prosthetic device
Knee replacement surgery	1VG53LAPPA	Implantation of internal device, knee joint with bone autograft tri component prosthetic device
Knee replacement surgery	1VG53LAPPK	Implantation of internal device, knee joint with bone homograft tri component prosthetic device
Knee replacement surgery	1VG53LAPPN	Implantation of internal device, knee joint with synthetic material
Knee replacement surgery	1VG53LAPPQ	Implantation of internal device, knee joint with combined sources of tissue
Knee replacement surgery	1VG53LAPR	Implantation of internal device, knee joint uncemented partial component [e.g. tibial liner (insert) alone]
Open control of bleeding, drainage, removal of device and inspection, abdominal cavity	1OT13LA	Control of bleeding, abdominal cavity using open approach
Open control of bleeding, drainage, removal of device and inspection, abdominal cavity	1OT13LANP	Control of bleeding, Abdominal cavity using open approach and leaving packing in situ
Open control of bleeding, drainage, removal of device and inspection, abdominal cavity	1OT52LA	Drainage, abdominal cavity using open approach
Open control of bleeding, drainage, removal of device and inspection, abdominal cavity	1OT52LATS	Drainage, abdominal cavity using open (incisional) approach and leaving drainage tube in situ
Open control of bleeding, drainage, removal of device and inspection, abdominal cavity	1OT52MFSJ	Drainage, abdominal cavity using open approach with shunt terminating in circulatory system [e.g. LeVeen Shunt, Denver Shunt]

Open control of bleeding, drainage, removal of device and inspection, abdominal cavity	1OT55LANP	Removal of device, abdominal cavity of (surgical) packing using open approach
Open control of bleeding, drainage, removal of device and inspection, abdominal cavity	1OT55LASJ	Removal of device, abdominal cavity of shunt system [e.g. peritoneal-venous shunt]
Open control of bleeding, drainage, removal of device and inspection, abdominal cavity	1OT70LA	Incision NOS, abdominal cavity using open approach
Open control of bleeding, drainage, removal of device and inspection, abdominal cavity	2OT70LA	Inspection, abdominal cavity using open approach
Pacemaker insertion	1HB53LAJA	Implantation of internal device, epicardium of pacemaker/defibrillator leads using open [thoracotomy] approach
Pacemaker insertion	1HB54LAJA	Management of internal device, epicardium of pacemaker/defibrillator leads using open [thoracotomy] approach
Pacemaker insertion	1HB55LAJA	Removal of device, epicardium of pacemaker/defibrillator leads and open approach
Pacemaker insertion	1HB55LAJB	Removal of device, epicardium of electrode (e.g. pacemaker, defibrillation lead) using laser (extraction) and open approach
Pacemaker insertion	1HD53GRJA	Implantation of internal device, endocardium of pacemaker/defibrillator leads using percutaneous [transvenous] approach
Pacemaker insertion	1HD54GRJA	Management of internal device, endocardium of pacemaker/defibrillator leads using percutaneous [transvenous] approach
Pacemaker insertion	1HD55GQJB	Removal of device, endocardium of electrode (e.g. pacemaker, defibrillation lead) by laser (extraction) using percutaneous transluminal (arterial) approach
Pacemaker insertion	1HD55GRJA	Removal of device, endocardium of pacemaker/defibrillator leads using percutaneous (transvenous) approach
Pacemaker insertion	1HZ53GRFR	Implantation of internal device, heart NEC percutaneous transluminal [transvenous] approach or approach NOS cardiac resynchronization therapy pacemaker [CRT, CRT-P, Biventricular pacemaker]
Pacemaker insertion	1HZ53GRFS	Implantation of internal device, heart NEC percutaneous transluminal [transvenous] approach or approach NOS cardioverter/defibrillator [AICD]
Pacemaker insertion	1HZ53GRFU	Implantation of internal device, heart NEC percutaneous transluminal [transvenous] approach or approach NOS cardiac resynchronization therapy defibrillator [CRT-D, BiV-ICD]

Pacemaker insertion	1HZ53GRNK	Implantation of internal device, heart NEC percutaneous transluminal [transvenous] approach or approach NOS dual chamber rate responsive pacemaker [DVI, DDD, DDDR modes]
Pacemaker insertion	1HZ53GRNL	Implantation of internal device, heart NEC percutaneous transluminal [transvenous] approach or approach NOS fixed rate pacemaker [VOO mode]
Pacemaker insertion	1HZ53GRNM	Implantation of internal device, heart NEC percutaneous transluminal [transvenous] approach or approach NOS single chamber rate responsive pacemaker [VDD, VVD, VVI, AAI, VVIR, AAIR modes]
Pacemaker insertion	1HZ53GRNN	Implantation of internal device, heart NEC percutaneous transluminal [transvenous] approach or approach NOS temporary pacemaker
Pacemaker insertion	1HZ53HNFS	Implantation of internal device, heart NEC percutaneous approach (to tunnel subcutaneously) cardioverter/defibrillator [AICD]
Pacemaker insertion	1HZ53LAFR	Implantation of internal device, heart NEC open [thoracotomy] approach cardiac resynchronization pacemaker [CRT, CRT-P, Biventricular pacemaker]
Pacemaker insertion	1HZ53LAFS	Implantation of internal device, heart NEC open [thoracotomy] approach cardioverter/defibrillator [AICD]
Pacemaker insertion	1HZ53LAFU	Implantation of internal device, heart NEC open [thoracotomy] approach cardiac resynchronization therapy defibrillator [CRT-D, BiV-ICD]
Pacemaker insertion	1HZ53LAKP	Implantation of internal device, heart NEC open [thoracotomy] approach artificial heart
Pacemaker insertion	1HZ53LANK	Implantation of internal device, heart NEC open [thoracotomy] approach dual chamber rate responsive pacemaker [DVI, DDD, DDDR modes]
Pacemaker insertion	1HZ53LANL	Implantation of internal device, heart NEC open [thoracotomy] approach fixed rate pacemaker [VOO mode]
Pacemaker insertion	1HZ53LANM	Implantation of internal device, heart NEC open [thoracotomy] approach single chamber rate responsive pacemaker [VDD, VVD, VVI, AAI, VVIR, AAIR modes]
Pacemaker insertion	1HZ53LANN	Implantation of internal device, heart NEC open [thoracotomy] approach temporary pacemaker
Pacemaker insertion	1HZ53QANK	Implantation of internal device, heart NEC open subxiphoid approach dual chamber rate responsive pacemaker [DVI, DDD, DDDR modes]
Pacemaker insertion	1HZ53QANL	Implantation of internal device, heart NEC open subxiphoid approach fixed rate pacemaker [VOO mode]
Pacemaker insertion	1HZ53QANM	Implantation of internal device, heart NEC open subxiphoid approach single chamber rate responsive pacemaker [VDD, VVD, VVI, AAI, VVIR, AAIR modes]
Pacemaker insertion	1HZ53SYFR	Implantation of internal device, heart NEC combined open [thoracotomy] approach and percutaneous transluminal [transvenous] approach cardiac resynchronization therapy pacemaker [CRT, CRT-P, Biventricular pacemaker]
Pacemaker insertion	1HZ53SYFS	Implantation of internal device, heart NEC combined open [thoractomy] approach and percutaneous transluminal [transvenous] approach cardioverter/defibrillator [AICD]
Pacemaker insertion	1HZ53SYFU	Implantation of internal device, heart NEC combined open [thoracotomy] approach and percutaneous transluminal

		[transvenous] approach cardiac resynchronization therapy defibrillator [CRT-D, BiV-ICD]
Pacemaker insertion	1HZ55GPFR	Removal of device, heart NEC percutaneous transluminal approach cardiac resynchronization therapy pacemaker [CRT, CRT-P, Biventricular pacemaker]
Pacemaker insertion	1HZ55GPFS	Removal of device, heart NEC percutaneous transluminal approach cardioverter/defibrillator [AICD]
Pacemaker insertion	1HZ55GPFU	Removal of device, heart NEC percutaneous transluminal approach cardiac resynchronization therapy defibrillator [CRT-D, BiV-ICD]
Pacemaker insertion	1HZ55GPNK	Removal of device, heart NEC percutaneous transluminal approach dual chamber rate responsive pacemaker
Pacemaker insertion	1HZ55GPNL	Removal of device, heart NEC percutaneous transluminal approach fixed rate pacemaker
Pacemaker insertion	1HZ55GPNM	Removal of device, heart NEC percutaneous transluminal approach single chamber rate responsive pacemaker
Pacemaker insertion	1HZ55LAFR	Removal of device, heart NEC open approach (e.g. sternotomy) cardiac resynchronization therapy pacemaker [CRT, CRT-P, Biventricular pacemaker]
Pacemaker insertion	1HZ55LAFS	Removal of device, heart NEC open approach (e.g. sternotomy) cardioverter/defibrillator [AICD]
Pacemaker insertion	1HZ55LAFU	Removal of device, heart NEC open approach (e.g. sternotomy) cardiac resynchronization therapy defibrillator [CRT-D, BiV-ICD]
Pacemaker insertion	1HZ55LAKP	Removal of device, heart NEC open approach (e.g. sternotomy) artificial heart
Pacemaker insertion	1HZ55LANK	Removal of device, heart NEC open approach (e.g. sternotomy) dual chamber rate responsive pacemaker
Pacemaker insertion	1HZ55LANL	Removal of device, heart NEC open approach (e.g. sternotomy) fixed rate pacemaker
Pacemaker insertion	1HZ55LANM	Removal of device, heart NEC open approach (e.g. sternotomy) single chamber rate responsive pacemaker
Pacemaker insertion	1HZ55QAFS	Removal of device, heart NEC open subxiphoid approach cardioverter/defibrillator [AICD]
Pacemaker insertion	1HZ55QANK	Removal of device, heart NEC open subxiphoid approach dual chamber rate responsive pacemaker
Pacemaker insertion	1HZ55QANL	Removal of device, heart NEC open subxiphoid approach fixed rate pacemaker
Pacemaker insertion	1HZ55QANM	Removal of device, heart NEC open subxiphoid approach single chamber rate responsive pacemaker
Pacemaker insertion	1YY54LAFR	Management of internal device, skin of surgically constructed sites of resynchronization pacemaker device using open (subcutaneous) approach
Pacemaker insertion	1YY54LAFS	Management of internal device, skin of surgically constructed sites of cardioverter or defibrillation device using open (subcutaneous) approach
Pacemaker insertion	1YY54LAFU	Management of internal device, skin of surgically constructed sites of resynchronization defibrillator device using open (subcutaneous) approach
Pacemaker insertion	1YY54LANM	Management of internal device, skin of surgically constructed sites of cardiac pacemaker battery/generator using open (subcutaneous) approach
Prostatectomy	1QT59BAAD	Destruction, prostate endoscopic per orifice [transurethral] approach using cryosurgery

Prostatectomy	1QT59BAAG	Destruction, prostate endoscopic per orifice [transurethral] approach using laser (with or without photosensitizing agent)
Prostatectomy	1QT59BAAW	Destruction, prostate endoscopic per orifice [transurethral] approach using radiofrequency
Prostatectomy	1QT59BAAZ	Destruction, prostate endoscopic per orifice [transurethral] approach using ultrasound device
Prostatectomy	1QT59BACG	Destruction, prostate endoscopic per orifice [transurethral] approach using microwave device
Prostatectomy	1QT59BAGX	Destruction, prostate endoscopic per orifice [transurethral] approach using device NEC
Prostatectomy	1QT59CAAZ	Destruction, prostate per orifice [transrectal] approach using ultrasound device
Prostatectomy	1QT59CACG	Destruction, prostate per orifice [transrectal] approach using microwave device
Prostatectomy	1QT59HAAD	Destruction, prostate percutaneous [transperineal] approach using cryosurgery
Prostatectomy	1QT59HAAG	Destruction, prostate percutaneous [transperineal] approach using laser (with or without photosensitizing agent)
Prostatectomy	1QT59HAAW	Destruction, prostate percutaneous [transperineal] approach using radiofrequency
Prostatectomy	1QT59HACG	Destruction, prostate percutaneous [transperineal] approach using microwave device
Prostatectomy	1QT59HAX7	Destruction, prostate percutaneous [transperineal] approach using chemical cautery [e.g. ethanol]
Prostatectomy	1QT59JAAQ	Destruction, prostate external approach using electromagnetic field [for activation of implanted thermal rods]
Prostatectomy	1QT87BAAG	Excision partial, prostate endoscopic per orifice [transurethral] approach Using laser NEC
Prostatectomy	1QT87BAAK	Excision partial, prostate endoscopic per orifice approach (transurethral) Using loop electrode
Prostatectomy	1QT87BAGX	Excision partial, prostate endoscopic per orifice approach [transurethral] using device NEC
Prostatectomy	1QT87DAGX	Excision partial, prostate endoscopic [laparoscopic] approach using device NEC
Prostatectomy	1QT87PBGX	Excision partial, prostate open perineal approach using device NEC
Prostatectomy	1QT87PKGX	Excision partial, prostate open retropubic approach using device NEC
Prostatectomy	1QT87QZAG	Excision partial, prostate open transvesical approach Using laser NEC
Prostatectomy	1QT87QZGX	Excision partial, prostate open transvesical approach [suprapubic] using device NEC
Prostatectomy	1QT91DA	Excision radical, prostate, using endoscopic (laparoscopic) approach
Prostatectomy	1QT91PB	Excision radical, prostate using open perineal approach
Prostatectomy	1QT91PK	Excision radical, prostate using open retropubic approach
Removal of appendix	1NV89DA	Excision total, appendix using endoscopic [laparoscopic] approach
Removal of appendix	1NV89LA	Excision total, appendix using open approach

Removal of gallbladder	1OD89DA	Excision total, gallbladder endoscopic [laparoscopic] approach without extraction (of calculi) cholecystectomy alone
Removal of gallbladder	1OD89DTAG	Excision total, gallbladder endoscopic [laparoscopic] approach with extraction (of calculi) from bile ducts using laser probe
Removal of gallbladder	1OD89DTAM	Excision total, gallbladder endoscopic [laparoscopic] approach with extraction (of calculi) from bile ducts using basket device
Removal of gallbladder	1OD89DTAS	Excision total, gallbladder endoscopic [laparoscopic] approach with extraction (of calculi) from bile ducts using electrohydraulic probe
Removal of gallbladder	1OD89DTBD	Excision total, gallbladder endoscopic [laparoscopic] approach with extraction (of calculi) from bile ducts using balloon device
Removal of gallbladder	1OD89DTGX	Excision total, gallbladder endoscopic [laparoscopic] approach with extraction (of calculi) from bile ducts using device NEC [e.g. forceps, metal probe]
Removal of gallbladder	1OD89EC	Excision total, gallbladder endoscopic [laparoscopic] approach cholecystectomy with bile duct exploration and no stones extracted
Removal of gallbladder	1OD89LA	Excision total, gallbladder open approach without extraction of calculi cholecystectomy alone
Removal of gallbladder	1OD89SMAG	Excision total, gallbladder open approach with extraction (of calculi) from bile ducts using laser probe
Removal of gallbladder	1OD89SMAM	Excision total, gallbladder open approach uwith extraction (of calculi) from bile ducts sing basket device
Removal of gallbladder	1OD89SMAS	Excision total, gallbladder open approach with extraction (of calculi) from bile ducts using electrohydraulic probe
Removal of gallbladder	1OD89SMBD	Excision total, gallbladder open approach with extraction (of calculi) from bile ducts using balloon device
Removal of gallbladder	1OD89SMGX	Excision total, gallbladder open approach with extraction (of calculi) from bile ducts using device NEC [e.g. forceps, metal probe]
Removal of gallbladder	1OD89TP	Excision total, gallbladder open approach cholecystectomy with bile duct exploration and no stones extracted
Repair of nasal cartilage and/or nose	1ES80LAXXA	Repair, nasal cartilage using autograft [e.g. cartilage, skin]
Repair of nasal cartilage and/or nose	1ES80LAXXE	Repair, nasal cartilage using local flap [e.g. alar cartilage rotation or transposition flap]
Repair of nasal cartilage and/or nose	1ES80LAXXG	Repair, nasal cartilage using pedicled flap
Repair of nasal cartilage and/or nose	1ES80LAXXK	Repair, nasal cartilage using homograft [e.g. cartilage]
Repair of nasal cartilage and/or nose	1ES80LAXXN	Repair, nasal cartilage using synthetic tissue [e.g. silastic sheath]
Repair of nasal cartilage and/or nose	1ES80LAXXQ	Repair, nasal cartilage using combined sources of tissue [e.g. graft and synthetic tissue]
Repair of nasal cartilage and/or nose	1ET80LA	Repair, nose without graft/implant using intranasal approach
Repair of nasal cartilage and/or nose	1ET80LAPM	Repair, nose with prosthetic implant using intranasal approach
Repair of nasal cartilage and/or nose	1ET80LAPMA	Repair, nose with prosthetic implant and autograft using intranasal approach

Repair of nasal cartilage and/or nose	1ET80LAXXA	Repair, nose with autograft using intranasal approach
Repair of nasal cartilage and/or nose	1ET80LAXXK	Repair, nose with homograft using intranasal approach
Repair of nasal cartilage and/or nose	1ET80WK	Repair, nose without graft/implant using columellar incision approach
Repair of nasal cartilage and/or nose	1ET80WKPM	Repair, nose with prosthetic implant using columellar incision approach
Repair of nasal cartilage and/or nose	1ET80WKPMA	Repair, nose with prosthetic implant and autograft using columellar incision approach
Repair of nasal cartilage and/or nose	1ET80WKXXA	Repair, nose with autograft using columellar incision approach
Repair of nasal cartilage and/or nose	1ET80WKXXK	Repair, nose with homograft using columellar incision approach
Repair of retina	1CN59LAAD	Destruction, retina using cryoprobe [cryotherapy]
Repair of retina	1CN59LAAG	Destruction, retina using laser
Repair of retina	1CN59LAGX	Destruction, retina using device NEC
Repair of retina	1CN59LAGY	Destruction, retina using multiple devices
Sterilization	1RF51DAAL	Occlusion, fallopian tube endoscopic [laparoscopic] approach using bipolar electrode
Sterilization	1RF51DAFA	Occlusion, fallopian tube endoscopic [laparoscopic] approach using band [ring]
Sterilization	1RF51DAFF	Occlusion, fallopian tube endoscopic [laparoscopic] approach using clips [e.g. plastic]
Sterilization	1RF51DALV	Occlusion, fallopian tube endoscopic [laparoscopic] approach using ligature (and transection or resection)
Sterilization	1RF51FJAL	Occlusion, fallopian tube endoscopic vaginal [culdoscopy, hysteroscopy] approach using bipolar electrode
Sterilization	1RF51FJFA	Occlusion, fallopian tube endoscopic vaginal [culdoscopy, hysteroscopy] approach using band [ring]
Sterilization	1RF51FJFF	Occlusion, fallopian tube endoscopic vaginal [culdoscopy, hysteroscopy] approach using clips (e.g. plastic)
Sterilization	1RF51FJGE	Occlusion, fallopian tube endoscopic vaginal [culdoscopy, hysteroscopy] approach using coil [e.g. micro-insert]
Sterilization	1RF51FJLV	Occlusion, fallopian tube endoscopic vaginal [culdoscopy, hysteroscopy] approach using ligature (and transection or resection)
Sterilization	1RF51LAAL	Occlusion, fallopian tube open approach using bipolar electrode
Sterilization	1RF51LAFA	Occlusion, fallopian tube open approach using band [ring]
Sterilization	1RF51LAFF	Occlusion, fallopian tube open approach using clips (e.g. plastic)
Sterilization	1RF51LALV	Occlusion, fallopian tube open approach using ligature (and transection or resection)
Tonsillectomy	1FR78DAAB	Repair by decreasing size, tonsils and adenoids using (percutaneous) endoscopic approach and scraping device (e.g. microdebrider)
Tonsillectomy	1FR89LA	Excision total, tonsils and adenoids tonsillectomy alone using device NEC
Tonsillectomy	1FR89LAAK	Excision total, tonsils and adenoids tonsillectomy alone using snare
Tonsillectomy	1FR89WJ	Excision total, tonsils and adenoids tonsillectomy with Adenoidectomy using device NEC

Tonsillectomy	1FR89WJAK	Excision total, tonsils and adenoids tonsillectomy with Adenoidectomy using snare

Appendix 1: Identification of HIV/AIDs and Alcohol Use Disorders

HIV/AIDS and alcohol use disorders will be identified using the CVSD based on ICD-10 diagnostic codes.

Cause Of Death	ICD-10 Codes
HIV/AIDS	B20-B24
Alcohol Related Diseases	F10, I426, K70, K85, K86.0, X45

Appendix 1: Identification of Covariates

ICD-10 diagnostic codes specific to comorbidities that will be used as covariates.

Comorbidity	ICD-9 Diagnostic Code	ICD-10 Diagnostic Code
Alcohol Related Diagnosis	291, 303, 305.0, 425.5, 571.0-571.3, 577.0-577.1, 860	F10.0, G31.2, G62.1, G72.1, I42.6, K29.2, K70.1, K70.4, K70.9, K86.0, K70.2, K70.3, X45, Z50.2, Z72.1, Z71.4, Z86.40, T51, Y15, Y91, K70, K85.2, T51.9
Chronic Lung Disease	470-478, 490-519	J45-46[26]
Chronic liver disease	456.1, 456.2, 070, 572.2, 572.3, 572.4, 572.8, 573, 782.4, V026, 275.0, 275.1, 789.1, 789.5, 571	K70.2, K70.3, K72.1, K74, B16-B19, I85, R17, R18, R16.0, R16.2, B94.2, Z22.50, Z22.5, E83.10, K71.3, K71.4, K71.5, K71.7, K72.9, K73, K75.3, K75.4, K75.8, K75.9, K76, K77
Chronic Kidney Disease	403, 403.1, 403.9, 404, 404.1, 404.9, 585, 586, 588.8, 588.9, 250.4	E10.2 E11.2, E13.2, E14.2, I12, I13, N08, N18, N19
Fibromyalgia	729.1	M79.7
Heart Disease	413, 427.3, 412, 410, 411, 425, 5184, 514, 428	I20, I21, I22, I25, I47, I48.0, I48.1, I49, I46.0, I46.9, R001
Lower Back Pain	724.2	M40, M41, M43, M46, M47, M48, M51, M53, M54, M96, M99, Q76.21, S22, S23, S32, S33
Rheumatoid Arthritis	714.0	M05, M06, M08.0, M08.2-M08.4, M08.8, M08.9, M09, M12.0
Mental Health	290-319	F04-F09, F20-F54, F56-F99, X60-X84, Y28
Osteoarthritis	715.0	M15.0-M15.2, M15.4, M16-M19, M89.41-M89.43, M89.45-M89.46, M89.48
Opioid Poisoning	965, 965.09, E850.2	T40.0, T40.1, T40.2, T40.3, T40.4, T40.6
Post-Traumatic Pain	338	G89.11

Appendix 5: Subgroup analysis results

Stratification variable	Model	Point Estimate	95% Wald Confidence Limits		Point Estimate	95% Wald Confidence Limits	
		Yes			No		
By having hospitalization of opioid poisoning or opioid use disorder in the previous four years	Crude	0.83	0.78	0.89	0.81	0.67	0.99
	Adjusted	1.26	1.17	1.37	1.16	0.93	1.45
By having hospitalization of alcohol-related diagnosis in the previous four years	Crude	0.86	0.81	0.92	1.19	0.97	1.47
	Adjusted	1.27	1.18	1.37	1.09	0.84	1.40
By having hospitalization of mental health diagnosis in the	Crude	0.79	0.73	0.85	1.04	0.93	1.17
	Adjusted	1.23	1.13	1.35	1.35	1.18	1.55

previous four years							
By marital status		Single, Widowed, Divorced, Separated, or Unknown			Married		
	Crude	1.04	0.97	1.12	0.78	0.69	0.89
	Adjusted	1.33	1.23	1.45	1.02	0.87	1.19
By quintile for FSA median income within province		Lowest quintile			Highest quintile		
	Crude	0.86	0.69	1.07	0.91	0.81	1.01
	Adjusted	1.34	1.04	1.73	1.22	1.07	1.39
